# Nonparametric Estimation of Transition Intensities in Interval‐Censored Markov Multistate Models Without Loops

**DOI:** 10.1002/sim.70225

**Published:** 2025-08-15

**Authors:** Daniel Gomon, Hein Putter

**Affiliations:** ^1^ Mathematical Institute Leiden University Leiden the Netherlands; ^2^ Department of Biomedical Data Sciences Leiden University Medical Centre Leiden the Netherlands

**Keywords:** EM algorithm, interval censoring, Markov, multistate model, NPMLE, panel data

## Abstract

Interval‐censored multistate data is collected when the state of a subject is observed periodically. The analysis of such data using nonparametric multistate models was not possible until recently but is very desirable as it allows for more flexibility than its parametric counterparts. The single available result to date has some unique drawbacks. We propose a nonparametric estimator of the transition intensities for interval‐censored multistate data using an Expectation Maximization algorithm. The method allows for a mix of interval‐censored and right‐censored (exactly observed) transitions. A condition to check for the convergence of the algorithm is given. A simulation study comparing the proposed estimator to a consistent estimator is performed and shown to yield near identical estimates at smaller computational cost. A data set on the emergence of teeth in children is analyzed. Software to perform the analyses is publicly available.

**Abbreviations**
EM: expectation maximization
NPMLE: nonparametric maximum likelihood estimator

## 1 Introduction

Nonparametric estimators such as the Kaplan–Meier survival curve are very popular in the standard survival setting with right‐censored time to event data. Their popularity comes from their flexibility, as they do not require assumptions to be made on the underlying form of the hazard rate. The research in survival analysis is very focused on right‐censored data, where event times are either observed exactly or known to occur after a certain censoring time. Many real life scenarios however generate interval‐censored data, where event times are only known to lie between two observation times. Such data often arises in medical studies, where patients are observed periodically over an extended interval of time. Nonparametric estimators have also been derived for interval‐censored data. Examples and an overview of analysis techniques for interval‐censored data are given in Bogaerts et al. [1] An important quantity in this setting is Turnbull's nonparametric estimator for the survival function [2]. Due to the additional complexity of interval‐censored data, the estimator requires the use of an Expectation Maximization (EM) algorithm. It might therefore be desirable to negate the need to use interval‐censored techniques, for example by using imputation techniques to recover the “missing” event time or ignore the missingness mechanism altogether and using techniques for right‐censored data instead. These approaches can lead to biases and incorrect inference [3, 4]. It is therefore important to use appropriate techniques in the presence of interval‐censored data.

**FIGURE 1:**
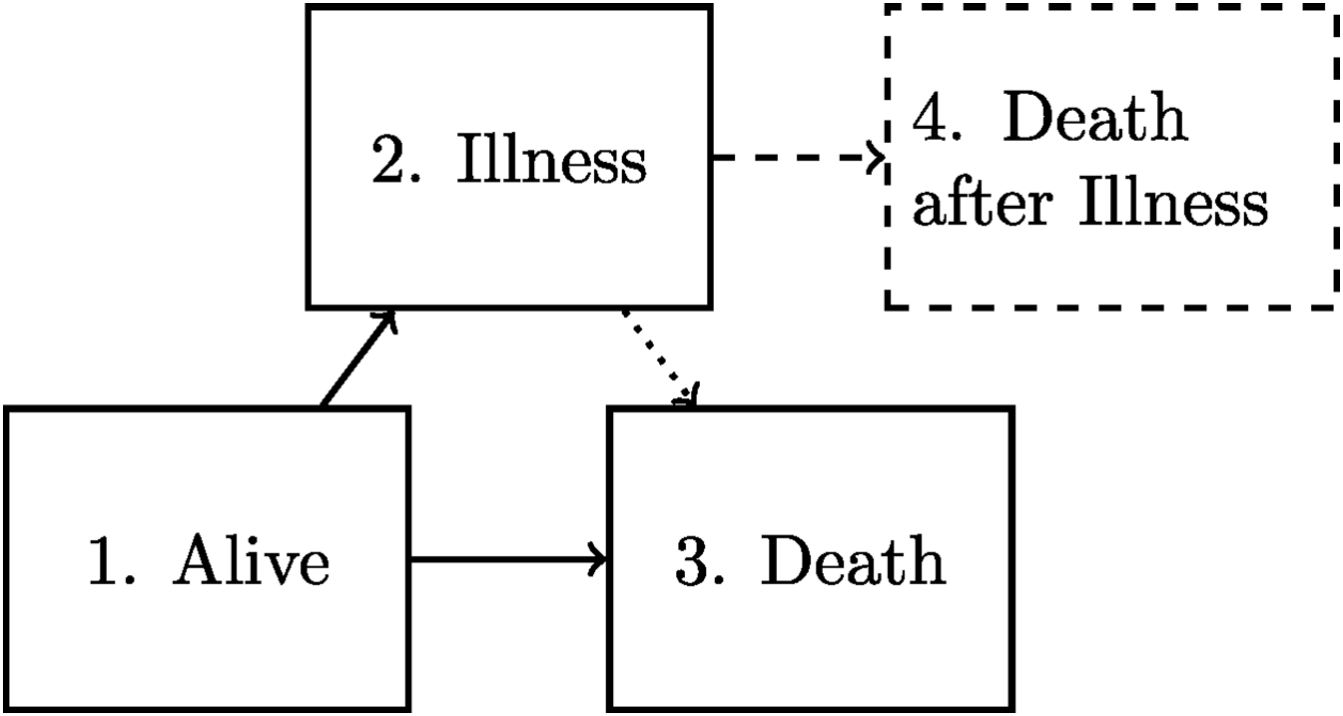
Graphical display of the (extended) illness‐death model. Extended: solid and dashed lines. Standard: solid and dotted lines.

The standard survival setting with a single time‐to‐event outcome is often insufficient to describe the data‐generating mechanism of a study. As an example, illness often precedes death in medical studies and the interest may lie in modeling both the time to death and time to illness. This situation can be well described using an (extended) illness‐death model (Figure 1), where the extended illness‐death model allows to distinguish between initially healthy subjects that have experienced illness and those who have not when calculating probabilities of experiencing death. These models are examples of a multistate model, a powerful generalization of the standard survival setting where the data is described using states (such as illness, death) and transitions (becoming ill, dying when healthy). Under the Markov assumption, the cumulative intensities can be nonparametrically estimated for multistate models with right‐censored data using the Nelson‐Aalen estimator and translated into transition probabilities using the Aalen‐Johanssen estimator [5]. Theory and practical applications are well described in many textbooks and tutorials [6, 7, 8]. In a multistate setting, data is also often interval‐censored when a subject's state is observed periodically. One setting which leads to such interval‐censored multistate data originates from studies based on *panel data*. Many demographic studies [9, 10] accrue one of more cohorts of subjects that are periodically assessed, often using questionnaires. Frequently these assessments (waves) are very regular in calendar time. However, when the analysis uses the subjects' ages as the time scale, such data typically leads to subject‐specific assessment times.

Contrary to the right‐censored setting, there are very few results that allow for the estimation of (cumulative) intensity functions in interval‐censored multistate models Chapter 5 of Cook and Lawless [6]. For specific multistate models, such as the (extended) illness‐death model, estimators are available in the nonparametric [11, 12] as well as the flexible parametric setting [13]. For general multistate models, some parametric results are available in the semi‐Markov framework [14, 15]. For Markov models, parametric results are restricted to the (time‐homogeneous) exponential distribution, allowing for (piecewise‐)constant intensity functions [16] and are available in the R package msm [17]. This time‐homogeneous approach is quite restrictive, yet numerical optimization is required to obtain estimates. For specific multistate models, the computational load can be reduced as analytic expressions are available for the components of the likelihood function. A flexible approach was proposed that allows for a mix of parametric and semiparametric intensity functions using P‐splines [18]. This method, however, relies heavily on the time‐homogeneous approach, as it approximates the chosen intensity functions by a fixed number of piecewise‐constant intensity functions and therefore does not estimate the parameters directly. Titman [19] also proposed a flexible approach, modeling the intensities using B‐splines. The estimates are then obtained by numerically solving the Kolmogorov forward equations, which can be computationally demanding in the presence of continuous covariates and experiences problems when the transition intensities have singularities. A nonparametric estimator for general interval‐censored multistate models was recently derived by Gu et al. [20, 21] using an EM algorithm with latent Poisson variables. The use of latent Poisson variables introduces some challenges, which we circumvent by taking a different approach.

In this article, we propose an Expectation Maximization algorithm to nonparametrically estimate the transition intensities in an interval‐censored Markov multistate model without loops. Without loops, the model does not allow for transitions to previously visited states. We extend the algorithm to allow for transitions to an arbitrary but fixed number of states to be observed at exact times, facilitating the analysis of a mix of interval‐ and right‐censored data. The notation, theory and EM algorithm are described in Section 2. In Section 2.5, the EM algorithm is described in detail and a method to determine convergence of the algorithm is given. The latent Poisson estimator [20] is described in Section 2.6 and used for comparison throughout the rest of the article. A simulation study is performed in Section 3, where the proposed estimator is compared with the latent Poisson [20] and the time‐homogeneous approach [17]. The three aforementioned methods are then used to analyze the Signal‐Tandmobiel study [22] on tooth emergence in children. The article is concluded by a discussion and recommendations for future research.

## 2 Methods

In this section, the notation for multistate models and data is introduced, as well as the proposed estimator. For further details on multistate modeling, the reader is referred to Cook and Lawless [6] and Andersen and Ravn [7].

### 2.1 Theory

Let X(t) represent a time nonhomogeneous Markov multistate model with states in ℋ={1,…,H} and denote the history (filtration) of the process up until time t as ${ℱ}_{t}=\{X(u);0\leq u\leq t\}$. Not all transitions between states in ℋ will be possible, we therefore let 𝒱={(g,h)∈ℋ×ℋ;direct transitiong→his possible} denote the set of possible direct transitions. We assume the model has no loops, implying the associated directed graph (see Figure 1) is acyclic. Without this assumption, a nonparametric estimator cannot be consistent as loops introduce uncertainty in the number of times a state is visited. Since we make no assumptions on the form of the intensities, we cannot compensate for this. For (g,h)∈𝒱 let the counting process $N_{gh}(t)$ denote the number of g→h transitions in [0,t]. As the counting processes for all transitions contain complete information on the process history, it can also be represented as ${ℱ}_{t}=\{N_{gh}(u);(g,h)\in 𝒱,0\leq u\leq t\}$.

The transition intensity, describing the instantaneous risk of a transition from state g to h with (g,h)∈𝒱 is given by:

$$\begin{array}{ll} \alpha_{gh}(t) & =\underset{dt↓0}{\lim}\frac{\mathbb{P}(X(t+dt)=h\;|\;X(t)=g,{ℱ}_{t-})}{dt}\\ & =\underset{dt↓0}{\lim}\frac{\mathbb{P}(X(t+dt)=h\;|\;X(t)=g)}{dt} \end{array}$$

where the second equality holds due to the Markov assumption. The cumulative transition intensity is then simply the integral $A_{gh}(t)=\int_{0}^{t}\alpha_{gh}(s)ds$. The process is only at risk of a transition at times when said transition can occur, therefore we also consider the transition intensity process:

$$\begin{array}{l} \lambda_{gh}(s)=\alpha_{gh}(s)Y_{g}(s) \end{array}$$

with $Y_{g}(s)=1\{X(s-)=g\}$ the at‐risk indicator for transitions out of state g and s− the time just before s. The intensity process is zero whenever the process is not at risk of a transition. Let C denote a right‐censoring time after which the state of the process is no longer known. For interval‐censored data, this is equal to the last time at which the state of the process is known. Additionally, we make the assumption of independent censoring:

$$\begin{array}{ll} \alpha_{gh}(t) & =\underset{dt↓0}{\lim}\frac{\mathbb{P}(X(t+dt)=h\;|\;X(t)=g,C>t)}{dt}\\ & =\underset{dt↓0}{\lim}\frac{\mathbb{P}(X(t+dt)=h\;|\;X(t)=g)}{dt} \end{array}$$

In other words, the knowledge that the process is currently uncensored does not influence the intensities.

Determining the probability of transitioning from one state to another is a vital component of multistate modeling. Letting 0≤s≤t, this quantity is given by the transition probabilities:

$$\begin{array}{l} P_{gh}(s,t)=\mathbb{P}(X(t)=h\;|\;X(s)=g) \end{array}$$

We gather the cumulative intensities and transition probabilities into H×H matrices $A(t)=\{A_{gh}(t);g,h\in ℋ\}$ with diagonal entries $A_{gg}(t)=-\sum_{h\neq g}A_{gh}(t)$ and $P(s,t)=\{P_{gh}(s,t);g,h\in ℋ\}$. For Markov multistate models, the Chapman‐Kolmogorov equations allow us to relate the transition probabilities to the transition intensities through product integration:

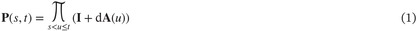

### 2.2 Data

We consider interval‐censored multistate data, where subjects i=1,…,n are observed to be in state $x_{ij}\in ℋ$ at random (or possibly fixed) subject‐specific visit times $T_{ij}$ with $j=1,\ldots ,n_{i}$. We denote $t_{ij}$ for the observed values of $T_{ij}$. Following Section 5.5 of Cook and Lawless [6] we assume that the subject observation times follow a conditionally independent visit process:

$$\begin{array}{l} T_{ij}⊥\{X_{i}(s),s>t_{i,j-1}\}\;|\;{ℱ}_{t_{i,j-1},i} \end{array}$$

with ${ℱ}_{t,i}$ the process history of individual i up until time t. This means that the next visit time must not depend on the state of the process since the last visit time, but can depend on all information available at the last visit time.

The observed set of information for subject i is then given by:

(2) $$\begin{array}{l} {𝕏}_{i}^{O}=\{(x_{i0},t_{i0}),(x_{i1},t_{i1}),\ldots ,(x_{in_{i}},t_{in_{i}})\} \end{array}$$

For each subject, we therefore observe realizations $X_{i}(t)$ of X(t) such that $X_{i}(t_{ij})=x_{ij}$. The observed data likelihood for interval‐censored multistate data is then given by:

(3) $$\begin{array}{l} L^{O}=\overset{n}{\underset{i=1}{\prod }}\overset{n_{i}}{\underset{j=1}{\prod }}P_{x_{i,j-1}x_{ij}}(t_{i,j-1},t_{ij}) \end{array}$$

**FIGURE 2:**
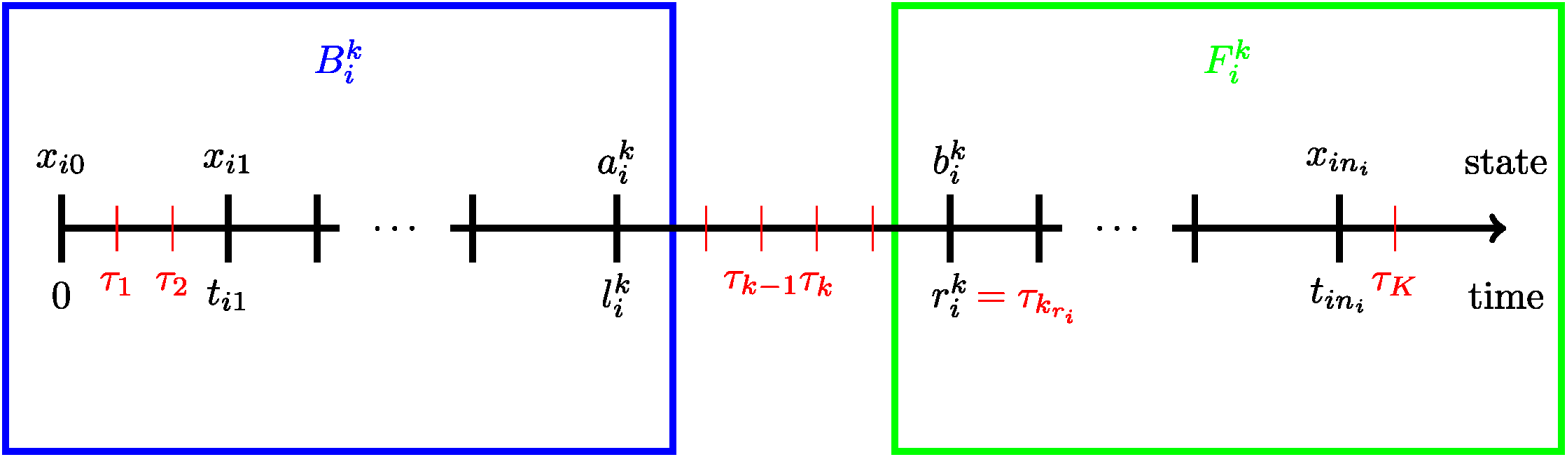
Graphical representation of data notation for a single subject i and fixed k∈{1,…,K}.

Let $0=\tau_{0}<\tau_{1}<\cdots <\tau_{K}<\infty$ be the sorted unique time points of the set $\{t_{ij}$; i=1,…,n, $j=1,\ldots ,n_{i}$}, and let $𝒯=\{\tau_{k};k=1,\ldots ,K\}$. We shall refer to an interval $\left(\tau_{k-1},\tau_{k}\right]$ for an arbitrary k as a *bin*. Assume that asymptotically the distance between unique observation times becomes arbitrarily small: $\lim_{n\to \infty }\max_{k}|\tau_{k}-\tau_{k-1}|=0$. Define for each subject i and state g the at risk indicator $Y_{g,i}(s)=1\{X_{i}(s-)=g\}$, indicating whether subject i is in state g just before time s and let $N_{gh,i}(t)$ count the number of transitions g→h of subject i in [0,t]. Note that the true state is only known at the observation times of the subjects. The value of $Y_{g,i}(s)$ is therefore only known right after the observation times. The number of transitions may be known if only a single path could have been taken by subject i, but this is not often the case. Similarly, we define $\lambda_{gh,i}(t)=\alpha_{gh}(t)Y_{g,i}(t)$ and let $dN_{gh,i}(t)$ be the observed jumps in [t,t+dt) such that $\mathbb{P}(dN_{gh,i}(t)=1\;|\;{ℱ}_{t-,i})=d\Lambda_{gh,i}(t)$. For fixed k∈{1,…,K} we define:

$$\begin{array}{l} l_{i}^{k}=\underset{j=1,\ldots ,n_{i}}{\max}\{t_{ij};t_{ij}\leq \tau_{k-1}\},r_{i}^{k}=\underset{j=1,\ldots ,n_{i}}{\min}\{t_{ij};t_{ij}\geq \tau_{k}\} \end{array}$$

so that $\left(l_{i}^{k},r_{i}^{k}\right]$ is the observational interval of subject i containing $\left(\tau_{k-1},\tau_{k}\right]$. Additionally, let $a_{i}^{k}=X_{i}(l_{i}^{k})$ and $b_{i}^{k}=X_{i}(r_{i}^{k})$ be the corresponding states occupied at the ends of this observational interval. We define $B_{i}^{k}:=\{X_{i}(t_{ij}),t_{ij}\leq l_{i}^{k}\}$ and $F_{i}^{k}:=\{X_{i}(t_{ij}),t_{ij}\geq r_{i}^{k}\}$ as the past and future observations relative to the interval $\left[l_{i}^{k},r_{i}^{k}\right]$. A graphical representation of this notation can be found in Figure 2.

### 2.3 EM Algorithm

In this section, we will nonparametrically estimate the cumulative transition intensities $A_{gh}(t)$ for interval‐censored multistate data using the Expectation Maximization (EM) algorithm [23]. The EM algorithm is an iterative procedure used to find maximum likelihood estimates in the presence of missing data. In the case of interval‐censored data, the exact transition times between states are missing for individuals, warranting the use of this algorithm.

#### 2.3.1 Complete‐Data Likelihood

To use the EM algorithm, we must first determine the complete data likelihood. Suppose we observe all transitions for all subjects and therefore know the exact transition times $t_{ij}^{*}$ for $j=1,\ldots ,f_{i}$, with $f_{i}$ the number of transitions made by subject i. For each subject i, we then have the following set of observations:

$$\begin{array}{l} {𝕏}_{i}^{C}=\{(s_{i0},t_{i0}^{*}),(s_{i1},t_{i1}^{*}),\ldots ,(s_{if_{i}},t_{if_{i}}^{*})\} \end{array}$$

with $X_{i}(t_{ij}^{*})=s_{ij}$. Under the assumption of independent censoring, the complete data likelihood contribution for a single subject is given by the canonical multinomial extension of the univariate likelihood (Section IV.4.1.5 of Andersen et al. [24]):

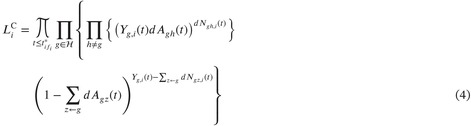

where $\sum_{z\leftarrow g}$ represents the sum over all states z that can be reached directly (in a single transition) from g. This has the interpretation of conditionally independent multinomially distributed numbers of jumps from each state g, such that $\left(dN_{gh}(t):h\neq g)∼\text{Mult}(Y_{g,i}(t),dA_{gh}(t):h\neq g\right)$ given ${ℱ}_{t-,i}$. For natural phenomena, it is reasonable to assume that the cumulative intensities are continuous. The product integral of the second term in Equation (4) can then be approximated by an exponent, leading to the following approximation of the contribution to the likelihood [6, 24]:

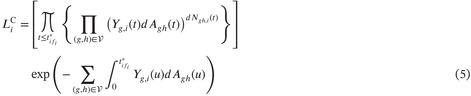

This can then be interpreted as a “Poisson” approximation of the canonical multinomial likelihood such that $\left(dN_{gh,i}(t):h\neq g)∼\text{Pois}(Y_{g,i}(t)dA_{gh}(t):h\neq g\right)$ given ${ℱ}_{t-,i}$. Although it is possible to consider Equation (4) for the EM‐algorithm, the use of the “Poisson” approximation (Equation 5) yields an estimator that is both easier to calculate and interpret. We therefore continue with Equation (5), and with a slight abuse in naming refer to this likelihood as the “multinomial” complete data likelihood. The resulting estimator using the canonical multinomial extension (Equation 4) as well as for a “multinoulli” approximation is found in Appendix B.

The log likelihood contribution for a single subject is then:

(6) $$\begin{array}{ll} \ell_{i}^{C} & :=\log(L_{i}^{C})=\int_{0}^{t_{if_{i}}^{*}}\underset{(g,h)\in 𝒱}{\sum }\left[\log\left(dA_{gh}(t)Y_{g,i}(t)\right)dN_{gh,i}(t)\right.\\ & \;\left.-dA_{gh}(u)Y_{g,i}(u)du\right] \end{array}$$

In the complete‐data setting, this expression will be maximized when the cumulative intensities are step functions with (possible) jumps at observed transition times. Although the exact transition times are not observed in the interval‐censored setting, the NPMLE in the two‐state survival setting has been shown to only have increments between some of the unique observation times [2]. Later, a similar result was found for the progressive three state model [11] and the illness‐death model [12]. The intervals where the cumulative intensities have increments are known as *support intervals*. In general, the total value of the increments within these support intervals can be estimated, but the locations within the support intervals cannot. Turnbull [2] therefore showed that the estimation problem could be reduced to the estimation of a right‐continuous step function, with jumps at the right endpoints of the support intervals, with the aim of estimating the jump sizes at these endpoints. Since the estimation problem for general interval‐censored multistate models is more complex, we cannot expect to achieve better results than these methods. We therefore simplify the problem to estimating cumulative transition intensity increments at the right‐endpoints of the bins, as the support intervals have been shown to depend completely on the unique observation times.

Following this line of reasoning, we restrict the estimation problem to cumulative intensity functions that can only make jumps at any of the unique observation times 𝒯, similarly to Gu et al. [20] This leads to an approximation of the complete‐data log‐likelihood, so that Equation (6) may be approximated by the sums:

(7) $$\begin{array}{ll} \ell_{i}^{C} & =\overset{K}{\underset{k=1}{\sum }}\underset{(g,h)\in 𝒱}{\sum }\left\{\log(\alpha_{gh}^{k}Y_{g,i}^{k})d_{gh,i}^{k}-\alpha_{gh}^{k}Y_{g,i}^{k}\right\}\\ & =\overset{K}{\underset{k=1}{\sum }}\underset{(g,h)\in 𝒱}{\sum }\left\{\log(\alpha_{gh}^{k})d_{gh,i}^{k}-\alpha_{gh}^{k}Y_{g,i}^{k}\right\} \end{array}$$

where $\alpha_{gh}^{k}=d{\bar{A}}_{gh}(\tau_{k})$, $Y_{g,i}^{k}={\bar{Y}}_{g,i}(\tau_{k})$ and $d_{gh,i}^{k}=d{\bar{N}}_{gh,i}(\tau_{k})$, with ${\bar{A}}_{gh},{\bar{Y}}_{g,i}$ and ${\bar{N}}_{gh,i}$ the cumulative intensity, at‐risk and counting processes in the simplified problem. These quantities then represent the jump of the cumulative intensity at $\tau_{k}$, presence in g at $\tau_{k-1}$ and transitions made between g and h at $\tau_{k}$ respectively. The second equality in Equation (7) holds because $d_{gh,i}^{k}=1$ implies $Y_{g,i}^{k}=1$, and $d_{gh,i}^{k}=1$ is only possible if $Y_{g,i}^{k}=1$. Now the approximate complete‐data log likelihood is obtained by summing over all subjects:

(8) $$\begin{array}{ll} \ell^{C}=\overset{n}{\underset{i=1}{\sum }}\ell_{i}^{C} & =\overset{K}{\underset{k=1}{\sum }}\underset{(g,h)\in 𝒱}{\sum }\left\{d_{gh}^{k}\log(\alpha_{gh}^{k})-\alpha_{gh}^{k}Y_{g}^{k}\right\} \end{array}$$

with $d_{gh}^{k}:=\sum_{i=1}^{n}d_{gh,i}^{k}$ and $Y_{g}^{k}:=\sum_{i=1}^{n}Y_{g,i}^{k}$. Although this is an approximation of the true complete‐data likelihood given in Equation (6), we will also refer to Equation (8) as the complete‐data likelihood throughout this article. It is important to keep in mind that this approximation is only reasonable if the assumption of diminishing distances between unique observation times holds.

Note that a support interval can consist of multiple bins and therefore the individual estimated jump sizes cannot be seen to represent the true increase in a single bin (although it is useful to think of them as such). Only the sum of the jumps over multiple bins that comprise a (to us unknown) support interval can be technically interpreted as an increment.

#### 2.3.2 E‐Step

In the E‐step of the EM algorithm, we calculate the expected value of the complete‐data likelihood using a guess for the jumps in the cumulative intensities of all transitions. We then determine new estimates for these jump sizes in the M‐step by maximizing the complete data likelihood and use these estimates as guesses in the next iteration, repeating this until convergence. Denote by $\alpha =(\alpha_{gh}^{k};g\neq h\in ℋ,k=1,\ldots ,K)$ the vector of jumps in the cumulative intensities, and with $\tilde{\alpha }=({\tilde{\alpha }}_{gh}^{k};g\neq h\in ℋ,k=1,\ldots ,K)$ the current estimate. Let $O=\{{𝕏}_{i}^{O};i=1,\ldots ,n\}$ denote the observed data and $C=\{{𝕏}_{i}^{C};i=1,\ldots ,n\}$ the complete data.

The main goal is to calculate the conditional expectation of the complete data log‐likelihood function:

(9) $$\begin{array}{ll} & 𝔼[\ell^{C}\;|\;O,\tilde{\alpha }]\\ & \;=\overset{K}{\underset{k=1}{\sum }}\underset{(g,h)\in 𝒱}{\sum }\left\{𝔼\left[d_{gh}^{k}\;|\;O,\tilde{\alpha }\right]\log(\alpha_{gh}^{k})-\alpha_{gh}^{k}𝔼\left[Y_{g}^{k}\;|\;O,\tilde{\alpha }\right]\right\} \end{array}$$

Note that this requires us to determine two conditional expectations. It can be shown that:

(10) $$\begin{array}{ll} d_{gh}^{k}(\tilde{\alpha }):=𝔼\left[d_{gh}^{k}\;|\;O,\tilde{\alpha }\right] & =\overset{n}{\underset{i=1}{\sum }}\frac{{\tilde{P}}_{a_{i}^{k},g}(l_{i}^{k},\tau_{k-1})\cdot {\tilde{\alpha }}_{gh}^{k}\cdot {\tilde{P}}_{h,b_{i}^{k}}(\tau_{k},r_{i}^{k})}{{\tilde{P}}_{a_{i}^{k},b_{i}^{k}}(l_{i}^{k},r_{i}^{k})} \end{array}$$

(11) $$\begin{array}{ll} Y_{g}^{k}(\tilde{\alpha }):=𝔼\left[Y_{g}^{k}\;|\;O,\tilde{\alpha }\right] & =\overset{n}{\underset{i=1}{\sum }}\frac{{\tilde{P}}_{a_{i}^{k},g}(l_{i}^{k},\tau_{k-1})\cdot {\tilde{P}}_{g,b_{i}^{k}}(\tau_{k-1},r_{i}^{k})}{{\tilde{P}}_{a_{i}^{k},b_{i}^{k}}(l_{i}^{k},r_{i}^{k})} \end{array}$$

with 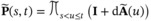 the product integral of the current estimates of the transition intensities. The derivation is found in Appendix section Interval‐censored multistate data.

#### 2.3.3 M‐Step

Having found the expected value of the likelihood function under the current estimates of the parameters, we need to maximize the resulting expected likelihood function. The jumps in the cumulative intensities are bounded, so the maximum must be found within a restricted optimization space. Luckily, the complete data log‐likelihood is a concave function and therefore we can use convex optimization theory [25]. The intensities $\alpha_{gh}^{k}$ represent conditional probabilities of making a transition in a single bin and therefore must be bounded by zero and one. Additionally, the total probability of leaving a state g in any bin k must also be smaller or equal than one. We therefore define the following optimization region:

$$\begin{array}{ll} C_{\alpha } & =\left\{\alpha_{gh}^{k},g\neq h\in ℋ,k=1,\ldots ,K;\alpha_{gh}^{k}\geq 0,\underset{h\leftarrow g}{\sum }\alpha_{gh}^{k}\leq 1\right\} \end{array}$$

Note that the two conditions $\alpha_{gh}^{k}\geq 0$ and $\sum_{h\leftarrow g}\alpha_{gh}^{k}\leq 1$ guarantee that $0\leq \alpha_{gh}^{k}\leq 1$. We show in Appendix Section Interval‐censored multistate data that the expected value of the complete data likelihood is maximized over the above region by the following expression:

(12) $$\begin{array}{ll} \alpha_{gh}^{k} & =\left\{\begin{array}{ll} \frac{d_{gh}^{k}(\tilde{\alpha })}{Y_{g}^{k}(\tilde{\alpha })},\; & \mu_{g}^{k}=0,\\ \frac{d_{gh}^{k}(\tilde{\alpha })}{\sum_{h\leftarrow g}d_{gh}^{k}(\tilde{\alpha })},\; & \mu_{g}^{k}>0 \end{array}\right. \end{array}$$

with

(13) $$\begin{array}{l} \mu_{g}^{k}=\max\left(0,\underset{h\leftarrow g}{\sum }\;\overset{k}{\underset{gh}{d}}(\tilde{\alpha })-\overset{k}{\underset{g}{Y}}(\tilde{\alpha })\right) \end{array}$$

Having found this expression, we can use the EM algorithm until a certain convergence criterion is met, see Algorithm 1.

### 2.4 Including Exactly Observed Transition Times

Until now we have considered the case of interval‐censored multistate data. In multistate models, there are often states for which the entry time into the state is exactly observed (or right‐censored). In the illness‐death model for example, it is unlikely that the exact time of the occurrence of a disease is known but the exact time of death is almost always known. These “exactly observed” states are oftentimes absorbing, but we do not have to make this assumption. In this section, we therefore consider the possibility of transitions into a fixed and known subset ℰ⊆ℋ to be observed at exact times, so that for an observation $\left(x_{ij},t_{ij}\right)$ with $x_{ij}\in ℰ$ this implies that the transition to $x_{ij}$ happened at $t_{ij}$. To derive the intensities with exactly observed states, we follow the same steps as in Section 2.3.

Suppose we observe subject i arriving in state h∈ℰ at time $t_{ij}$ for some $j\in \{1,\ldots ,n_{i}\}$. This means that the process must have been in a state g∈ℋ which allows for a direct transition to h right before time $t_{ij}$. Define ${ℛ}_{h}=\{g\in ℋ;\text{direct transition}\;g\to h\;\text{is possible}\}$ as the states which h∈ℋ is directly reachable from. As we assumed that the cumulative intensities have upward jumps only at times in 𝒯, we therefore know that the subject must have been in ${ℛ}_{h}$ at time $t_{ij}-$. The observed data likelihood is now given by:

(14) $$\begin{array}{ll} L_{E}^{O} & =\overset{n}{\underset{i=1}{\prod }}\overset{n_{i}}{\underset{j=1}{\prod }}{\left\{P_{x_{i,j-1}x_{ij}}(t_{i,j-1},t_{ij})\right\}}^{1-1\{x_{ij}\in ℰ\}}\\ & \;{\left\{\underset{m\in {ℛ}_{x_{ij}}}{\sum }P_{x_{i,j-1}m}(t_{i,j-1},t_{ij}-)dA_{mx_{ij}}(t_{ij})\right\}}^{1\{x_{ij}\in ℰ\}} \end{array}$$

as opposed to Equation (3). Note that $t_{ij}=\tau_{k}$ for some k∈{1,…,K}, therefore $t_{ij}-=\tau_{k-1}$. On the other hand, the complete data still consists of all (exact) transition times, therefore, the complete‐data likelihood (Equation 8) does not change. In the E‐step however, we now have additional information for the calculation of the expected values whenever a state in ℰ is observed. Note that this additional information improves the variance of the estimation procedure, as there is more certainty about the states a subject can reside in before an exact transition and fewer possible transitions can have occurred at said time. Having many exactly observed states therefore always makes the estimation procedure “easier” and more precise.

#### 2.4.1 E‐Step

To incorporate the newly available information, we re‐evaluate the quantities $d_{gh}^{k}(\tilde{\alpha })$ and $Y_{g}^{k}(\tilde{\alpha })$. We show in Appendix Section Interval Censored Multistate Data With Exactly Observed States that when $b_{i}^{k}\in ℰ$ the conditional expectations are instead given by:

(15) $$\begin{array}{ll} d_{gh,i}^{k}(\tilde{\alpha }) & :=𝔼\left[d_{gh,i}^{k}\;|\;O,\tilde{\alpha }\right]\\ & =\frac{{\tilde{P}}_{a_{i}^{k}g}(l_{i}^{k},\tau_{k-1})\cdot {\tilde{\alpha }}_{gh}^{k}\cdot \sum_{m\in {ℛ}_{b_{i}^{k}}}{\tilde{\alpha }}_{mb_{i}^{k}}^{k_{r_{i}}}{\tilde{P}}_{hm}(\tau_{k},\tau_{k_{r_{i}}-1})}{\sum_{m\in {ℛ}_{b_{i}^{k}}}{\tilde{\alpha }}_{mb_{i}^{k}}^{k_{r_{i}}}{\tilde{P}}_{a_{i}^{k}m}(l_{i}^{k},\tau_{k_{r_{i}}-1})} \end{array}$$

(16) $$\begin{array}{ll} Y_{g,i}^{k}(\tilde{\alpha }) & :=𝔼\left[Y_{g,i}^{k}\;|\;O,\tilde{\alpha }\right]\\ & =\frac{{\tilde{P}}_{a_{i}^{k}g}(l_{i}^{k},\tau_{k-1})\cdot \sum_{m\in {ℛ}_{b_{i}^{k}}}{\tilde{\alpha }}_{mb_{i}^{k}}^{k_{r_{i}}}{\tilde{P}}_{gm}(\tau_{k-1},\tau_{k_{r_{i}}-1})}{\sum_{m\in {ℛ}_{b_{i}^{k}}}{\tilde{\alpha }}_{mb_{i}^{k}}^{k_{r_{i}}}{\tilde{P}}_{a_{i}^{k}m}(l_{i}^{k},\tau_{k_{r_{i}}-1})} \end{array}$$

with $\tau_{k_{r_{i}}}:=r_{i}^{k}$, $d_{gh}^{k}(\tilde{\alpha })=\sum_{i=1}^{n}d_{gh,i}^{k}(\tilde{\alpha })$ and $Y_{g}^{k}(\tilde{\alpha })=\sum_{i=1}^{n}Y_{g,i}^{k}(\tilde{\alpha })$. When $b_{i}^{k}\notin ℰ$ the conditional expectations are still given by Equations (10) and (11).

#### 2.4.2 M‐Step

For the M‐step, we would like to maximize the expected complete data likelihood function. Fortunately, nothing changes with respect to Section 2.3.3, therefore we can still utilize Equation (12) to update our estimate of the intensities.

### 2.5 EM Algorithm

The theory above suggests a way for the computation of the nonparametric maximum likelihood estimate of the intensities. An algorithm displaying the necessary steps to arrive at an estimate is given in Algorithm 1.

ALGORITHM 1 **Calculation of NPMLE of intensities in a MSM without loops.**

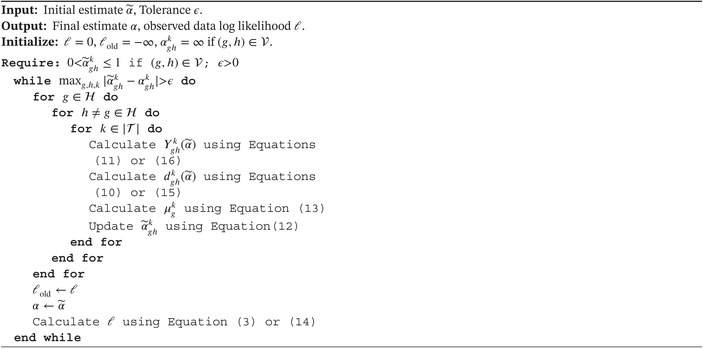

ALGORITHM 2 **Determine whether a (local) maximum has been reached.**

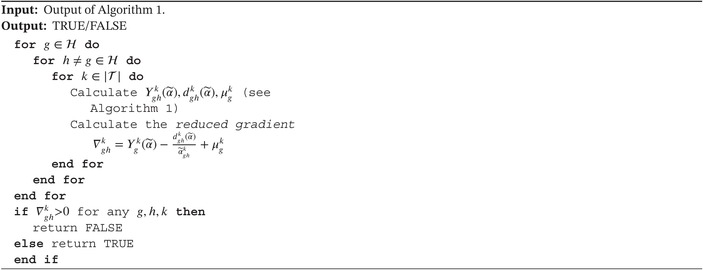

Algorithm 1 uses an intensity convergence criterion and therefore terminates when the largest change in any intensity is smaller than the tolerance. A different criterion could also be used, such as a likelihood convergence criterion: $|L-L_{\text{old}}|>\epsilon$. Another approach can also be taken. For a convex optimization problem, whenever an estimate $\tilde{\alpha }$ satisfies the Karush–Kuhn–Tucker conditions (details in Appendix Section Interval‐Censored Multistate Data) we are guaranteed to have arrived at a (local) maximum of the expected complete‐data likelihood function (Section 5.5.3 of Boyd and Vandenberghe [25]). When this is the case, further iterations of the EM algorithm will not improve the objective function. This suggests a method to check whether the algorithm should be terminated, without keeping track of estimated quantities in previous iterations. Such a procedure is outlined in Algorithm 2. The reduced gradient $\nabla_{gh}^{k}$ has the interpretation of how much the objective function can be improved by changing the corresponding estimate. Note that the reduced gradient can be calculated during every iteration of Algorithm 1 at little cost. Although Algorithm 2 suggests checking whether the reduced gradient is greater than 0, it is also possible to stop when $\nabla_{gh}^{k}<\epsilon$ for all transitions and time bins.

### 2.6 Latent Poisson EM

Recently an EM approach for the NPMLE using latent Poisson variables has been developed [20], allowing for the inclusion of (time‐dependent) covariates and random effects through a semiparametric proportional hazards model. For comparison purposes, we consider their model without any covariates/random effects.

The main difference between their approach and ours is the complete data likelihood used to maximize the observed‐data likelihood (Equation 3). In Section 2.3.1, we took the multinomial likelihood approach as described in Section 2.2 of Cook and Lawless [6], whereas they take a latent Poisson approach. They also assume that the cumulative intensities only have jumps at the unique event times, but consider latent Poisson variables $W_{gh,i}^{k}$ for each bin instead of $d_{gh,i}^{k}$ as we do. These variables then indicate whether a certain transition in a bin could have happened (latently), and assign (non)‐zero mass if so. They then show that the observed data likelihood using latent Poisson variables is the same as Equation (3), therefore the observed‐data likelihood can be maximized using the Poisson full data likelihood:

(17) $$\begin{array}{ll} \ell_{P}^{C} & =\overset{n}{\underset{i=1}{\sum }}\left(\overset{K}{\underset{k=1}{\sum }}\underset{(g,h)\in 𝒱}{\sum }1\{\tau_{k}\leq t_{in_{i}}\}\right.\\ & \;\left.\left[W_{gh,i}^{k}\log(\alpha_{gh}^{k})-\alpha_{gh}^{k}-\log(W_{gh,i}^{k}!)\right]\right) \end{array}$$

Performing an E‐ and M‐step as we have, it is then possible to obtain an update rule for α, which is described in more detail in the Supporting Information Section B.

Although possible in the latent Poisson setting, exactly observed event times are not considered for an arbitrary number of states [21] referring to the inconsistency problems in mixed samples NPMLE estimation [26]. Instead, they consider only the situation with a single absorbing state, for which observation times are known exactly and model the transition intensities into this state using B‐splines. Due to the restriction to a single exactly observed state, we do not compare our model with the latent Poisson approach in the case of exactly observed states.

## 3 Simulation Study

To investigate the performance of our proposed estimator, we conduct a simulation study. Seeing as the latent Poisson EM estimator (Section 2.6) has been shown to be consistent [20], we focus on comparing our method to this estimator. Quantities of primary interest are the bias and variance of the estimator with respect to the cumulative intensities and the transition probabilities as well as computation speed.

### 3.1 Data Generation

We consider six scenarios in this simulation study. The aim of the scenarios is to investigate the effect of varying parameters of the data generation model. We first cover the parameters which are equal for all scenarios. We assume that all n subjects are observed at the beginning of the study (time 0) and that all observations are censored at the end of the study after 15 years. We consider n=100,300, and 500 subjects to assess the behavior of the estimator with increasing samples. For each scenario and each number of subjects n, we create N=1000 simulated data‐sets.

The following simulation parameters are scenario specific. We consider the illness‐death (ID) model, or the extended illness‐death (EID) model (see Figure 1). Observed states are simulated assuming underlying Weibull(λ,k) or Exponential(λ) distributions for the transition times, with Weibull probability density function $f(x;\lambda ,k)=\lambda kx^{k-1}e^{-\lambda x^{k}}$. The parameters were chosen so that the mean transition times were equal over all scenarios for comparable transitions. Subjects are either initially observed in the first (Alive) state or have an equal probability to start in the first or second (Illness) state. In some scenarios, death states are exactly observed. The interobservation times (time between two consecutive observations for a subject) follow a uniform distribution with varying parameters. Details are found in Table 1.

**TABLE 1:** Simulation parameters for the six considered scenarios.

Scenario	Model	Starting states	Exact states ℰ	(Inter‐)observation times	Transition distributions		
					1→2	1→3	2→3∨2→4
1	ID	1	∅	U[0,4.4]	Exp (0.1)	Exp (0.05)	Exp (0.1)
2	ID	{1,2}	∅	U[0,4.4]	Exp (0.1)	Exp (0.05)	Exp (0.1)
3	ID	1	∅	U[0,4.4]	$\text{Weib}\left(0.5,\frac{1}{\sqrt{5}}\right)$	$\text{Weib}\left(0.5,\frac{1}{\sqrt{10}}\right)$	$\text{Weib}\left(2,{\left(\frac{\Gamma (1.5)}{10}\right)}^{2}\right)$
4	EID	1	{3,4}	U[0,4.4]	Exp (0.1)	Exp (0.05)	Exp (0.1)
5	ID	{1,2}	∅	U[2.9,3.1]	Exp (0.1)	Exp (0.05)	Exp (0.1)
6	ID	{1,2}	∅	U[0,2.44] & U[0,7.33]	Exp (0.1)	Exp (0.05)	Exp (0.1)

In the last two scenarios (5 and 6), we investigate the effect of the observation schedule on the estimators. Scenario five represents the classical panel data schedule in chronological time. All subjects are scheduled to be observed once every three years, with their response time deviating at most 0.1 years in each direction uniformly. In the sixth scenario, two different observation schemes are considered, with either a uniform[0,2.44] or uniform[0,7.33] distribution for the inter‐observation times. This results in an average of 4 or 10 observations per subject in the 15 year observation period, as opposed to the average of 6 observations with uniform[0,4.4] inter‐observation times.

For each of the scenarios and n∈{100,300,500}, we fit three multistate models to each of the N=1000 simulated data sets. The three considered models are the multinomial and latent Poisson EM approaches as well as the time‐homogeneous approach [17] using the R [27] package msm. The time‐homogeneous approach assumes that transition intensities are constant over time per transition, which comes down to assuming exponential transition hazards. For scenarios 1,2,4, and 5, transition times are therefore generated under the time‐homogeneous assumption. The initial estimates for the EM algorithms are chosen as ${\tilde{\alpha }}_{gh}^{k}=\frac{1}{K}$ if (g,h)∈𝒱 and zero elsewhere and the algorithms are terminated when the change in all transition intensities is smaller than 0.001. The time‐homogeneous model is run with default parameters, letting the msm package determine initial estimates.

To obtain performance measures, we define the estimated cumulative intensities for the vth data set as ${\hat{A}}_{gh}^{v}(t)$ for all (g,h)∈𝒱 and transition probabilities ${\hat{P}}_{gh}^{v}(t)$ for any tuple (g,h). The true (oracle) values are denoted by $A_{gh}(t)$ and $P_{gh}(t)$. We then calculate the bias, variance and root mean squared error (RMSE) of the estimators over an equidistantly spaced partition of [0,15]. For the cumulative intensities, fixing (g,h)∈𝒱 and t∈{0,0.1,0.2,…,15} we calculate:

(18) $$\begin{array}{ll} \text{Bias}(t) & =\frac{\sum_{v=1}^{N}\left({\hat{A}}_{gh}^{v}(t)-A_{gh}(t)\right)}{N}\\ \mathrm{Var}(t) & =\frac{1}{N-1}\overset{N}{\underset{v=1}{\sum }}{\left({\hat{A}}_{gh}^{v}(t)-\frac{\sum_{v=1}^{N}{\hat{A}}_{gh}^{v}(t)}{N}\right)}^{2} \end{array}$$

and $\text{RMSE}(t)=\sqrt{\mathrm{Var}(t)+{\text{Bias}}^{2}(t)}$. Transition probabilities are calculated by taking the product integral (Equation 1) over the cumulative intensities.

### 3.2 Results

For clarity, we show only the results for n=500, but the rest of the findings is found in Supporting Information Section A.

#### 3.2.1 Scenario 1

**FIGURE 3:**
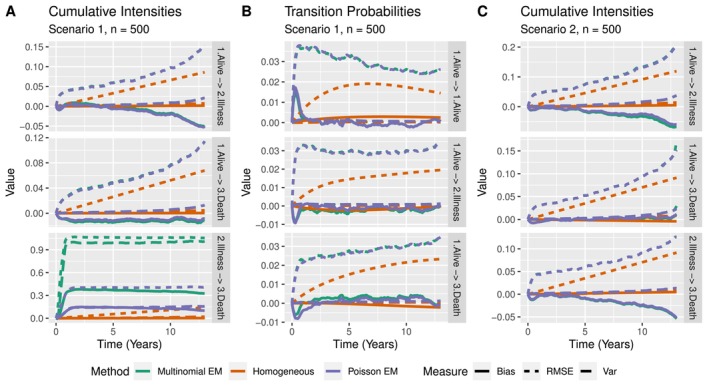
Bias, variance, and RMSE for n=500 subjects of A) cumulative intensities in scenario 1, B) transition probabilities in scenario 1, and C) cumulative intensities in scenario 2.

In the first scenario, we consider the simple illness‐death model with exponential transition hazards and all subjects starting in the alive state. Performance measures for the cumulative intensities of the three considered methods are found in Figure 3A. Overall, the time‐homogeneous model performs best as it is correctly specified in this scenario. The multinomial and latent Poisson approaches perform very similarly for transitions out of the alive state. For the transition from the illness state, the estimated cumulative intensity is very unstable in the first one to two years of the study for both EM approaches, but much more so for the multinomial approach. Ignoring the first two years however, the jumps in the cumulative intensities are recovered correctly as both the bias and variance curves remain relatively flat over time. As we are estimating conditional quantities (jumps in the cumulative intensities) instead of the marginal cumulative intensity function directly, we are interested in the slope of the performance curves rather than the value over time. The reason for the observed instability in the beginning of the study is the small number of subjects at risk of transitioning out of the illness state. The multinomial approach can then estimate a probability (jump in the cumulative intensity) close to one if only very few people are at risk and a subject makes a transition, explaining the jump of the variance curve to a value of around one. Similarly, as time progresses subjects will be absorbed in the death state, leaving less subjects for the estimation of intensities in all states. This explains the increase in the variability of the estimates over the study duration.

**FIGURE 4:**
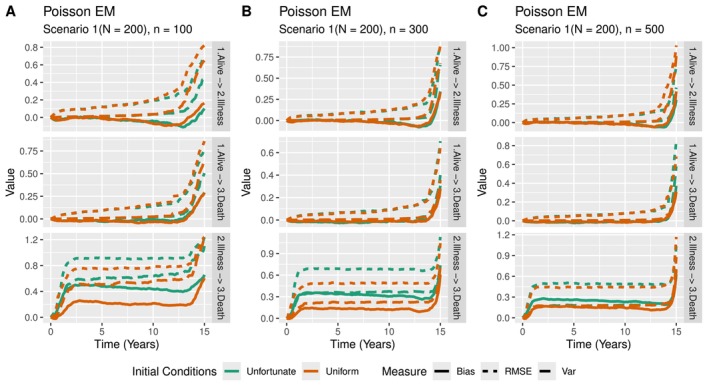
Bias, variance, and RMSE of cumulative intensities in scenario 1 with A) *n* = 100, B) *n* = 300, and C) *n* = 500 subjects in N=200 simulated data sets. Only Poisson EM is considered, with uniform and “unfortunate” initial conditions.

The latent Poisson approach cannot change the estimates of its intensities for the 2→3 transition before a subject has entered state 2 (see Supporting Information Section C). Because of this, the initial bias for the Poisson approach in this scenario seems smaller, but is actually completely determined by the initial estimates. As the initial estimates were chosen uniformly depending on the number of unique observation times, the variance also results purely from the difference in observation times between data sets. To illustrate this occurrence, we fit the Poisson EM model on the first 200 data‐sets of this scenario using “unfortunate” initial estimates. These “unfortunate” initial estimates were chosen such that $\sum_{k=1}^{K}\alpha_{gh}^{k}=1$ and $\sum_{k=1}^{\left\lfloor 0.1K\right\rfloor }\alpha_{gh}^{k}=0.9$ for all (g,h)∈𝒱. In other words, we assigned 90% of the initial “mass” to the first 10 percent of the bins. A comparison of the results for uniform and “unfortunate” initial estimates can be found in Figure 4. Clearly, changing the initial estimates significantly changes the performance of the method for the 2→3 transition, which is a very undesirable property.

Oftentimes, transition probabilities are of greater interest than cumulative intensities. As all subjects start in the (first) alive state in this scenario, we take a look at the bias, variance and RMSE when recovering the transition probabilities from the first state $P_{1h}(t)$ for h∈{1,2,3}. Note that by considering the transition probabilities only from the first state, we can negate the problem of the small risk set encountered when considering the cumulative intensities. The results can be found in Figure 3B. Unsurprisingly the time‐homogeneous approach performs best. The multinomial and Poisson approach do not differ by much, but the bias for the probability of arriving in state 3 (death) while starting in state 1 (alive) is smaller in early time points for the multinomial approach, contrary to the observations for the cumulative intensities. Note that subjects can arrive in state 3 through two different paths: either through illness (1→2→3) or directly (1→3). Since transition probabilities are derived from estimated intensity jumps, the multinomial estimates, though seemingly more biased, outperform the latent Poisson estimates at early time points, which rely solely on initial guesses. All in all, both the Poisson and multinomial approach lead to biased estimates of the cumulative intensities for different reasons. Great care must be taken when interpreting estimates, as small risk sets can lead to unbalanced estimates for the multinomial approach. As the bias for the Poisson approach depends on the initial conditions, the multinomial estimator is preferred in models where subjects cannot start in every nonabsorbing state.

#### 3.2.2 Scenario 2

The Poisson EM estimate has been shown to be consistent for the cumulative intensities [20], but only when all nonabsorbing states have a positive probability to be the initially observed state. In scenario 2, both nonabsorbing states are equally likely as starting states. We can therefore compare the performance of the multinomial estimator to a consistent estimator. Figure 3C presents a comparison on the recovery of the underlying cumulative intensities.

There is not much difference between the Poisson and multinomial approach, and both perform well compared to the time‐homogeneous approach. As the sample size n increases, the RMSE of the EM estimators decreases at the same rate and they converge to almost identical estimates (see Supporting Information Section A). It is therefore reasonable to assume that our proposed estimator is also consistent for the cumulative intensities when the nonabsorbing states are covered in the initial observation.

#### 3.2.3 Scenario 3

**FIGURE 5:**
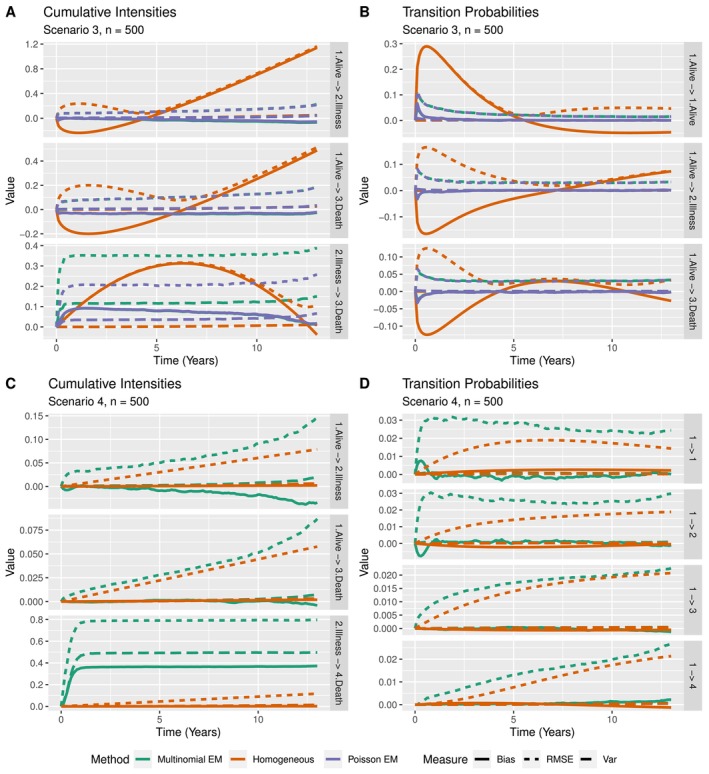
Bias, variance, and RMSE of cumulative intensities/transition probabilities for n=500 subjects in (A and B) scenario 3 and (C and D) scenario 4.

In the previous scenarios, the data was generated under the time‐homogeneous assumption. Now we take a look at how well the methods recover time varying intensities. The intensities for transitioning to illness and death were chosen to be decreasing functions of time, while the hazard from illness to death is increasing. In practice, this means that subjects are more likely to become ill or pass away at the beginning of the study, and subjects that are ill for a long time are more likely to die. Performance measures for the cumulative intensities and transition probabilities for n=500 can be found in Figure 5A,B.

As expected, the time‐homogeneous model does not recover the underlying form of the hazards at all. We observe the same problem due to the small risk sets as in Scenario 1, where the cumulative intensities seem to be more appropriately recovered by the latent Poisson approach, whereas the transition probabilities are recovered equally well by the two EM methods. All in all, the estimates are unreliable when only very few transitions are observed (early and late study times).

#### 3.2.4 Scenario 4

In this scenario, we consider the extended illness‐death model with both death states exactly observed. We compare only the multinomial EM method with the time‐homogeneous approach, as the latent Poisson approach was not worked out for this case. Note that the msm package allows for the inclusion of exactly observed states. The underlying hazards were therefore chosen to be exponential so that a comparison between the methods can be made. The results for n=500 is found in Figure 5C,D.

Both methods recover the transition probabilities from the initial state very well. The cumulative intensity estimate for the multinomial approach suffers from the low risk sets as before. The RMSE of the multinomial estimator is very similar to the well specified time‐homogeneous model. In conclusion, we recover the intensities well even in the case of exactly observed states.

#### 3.2.5 Scenario 5

Scenario 5 was considered to determine whether appropriate estimates could be recovered when classical panel data was considered in chronological time, resulting in no information being available on any subject for periods of at least 2.8 years at a time. As a result, the distance between two consecutive unique observation times $\left|\tau_{k}-\tau_{k-1}\right|$ is very large for some k. Figures summarizing the resulting performance of the estimators can be found in the Supporting Information Section A. Clearly, the cumulative intensities are not recovered well, with the estimator oscillating around the true value over time. It is not possible to recover the true form of the intensities from such an estimator, which exhibits both positive and negative bias over time. The negative bias occurs due to the absence of any information, which is then overcompensated by a sudden influx of observations, leading to positive bias. With time‐dependent intensities, this absence of information would make recovering the true form of the intensities even more difficult. The importance of the assumption that $\lim_{n\to \infty }\max_{k}|\tau_{k}-\tau_{k-1}|=0$ becomes apparent, as it is violated in this scenario.

#### 3.2.6 Scenario 6

The aim of scenario 6 is to determine whether the variance of the estimators becomes smaller as the largest distance between adjacent unique observation times $\max_{k}|\tau_{k}-\tau_{k-1}|$ becomes smaller. This is achieved by considering two observation schemes where subjects are observed less and more frequently over time. The two different observation schemes are compared on a sample of n=500 in the Supporting Information Section A. Not surprisingly, the variance decreases as subjects are observed more frequently for all methods. On average, more frequent observations guarantee that the observation intervals for subjects will be smaller, making the estimation procedure “easier”.

#### 3.2.7 Computation Speed

A critical attribute of a method is whether it can be used on real‐life data sets. The time required to obtain estimates plays a major role therein. Both EM approaches require the calculation of the same number of product integrals per iterations, and these calculations make up the largest part of the total computation time. Each iteration therefore takes roughly the same time for both approaches. To compare the two approaches, the mean number of iterations (and standard deviation) required to reach convergence over the N=1000 repeats in scenario 3 is shown in Table 2. The Poisson approach requires significantly more iterations to reach convergence than our proposed method and was therefore much slower in our experience. To put time in perspective, it took the multinomial EM algorithm on average 6.5 min to reach convergence on a data set with n=500 using a single core of the 2.6Ghz Intel Xeon Gold 6126 processor as opposed to 21 min for the Poisson algorithm.

**TABLE 2:** Mean number of iterations (standard deviation) required to reach convergence over the N=1000 data sets in scenario 3 for the two EM algorithms.

EM algorithm	Sample size		
	100	300	500
Multinomial	331(155)	474(162)	513(128)
Poisson	549(230)	684(202)	702(210)

The time‐homogeneous approach implemented in the msm package was extremely fast in the first three scenarios, taking less than a minute to run all simulations. In scenario 4 however, the homogeneous approach took about twice as long as the multinomial EM algorithm. This happened because msm uses the standard optimization routine in R to maximize the observed‐data likelihood, which is very slow when the (derivative of the) likelihood becomes hard to evaluate.

### 3.3 Key Findings

We summarize the findings from the simulation study:

- The Poisson estimator only produces interpretable estimates whenever subjects can start in all nonabsorbing states. If this is not the case, its estimates depend on the initial choice of intensities and the model should therefore be used with caution.
- The multinomial approach always produces interpretable estimates, but special care must be taken as small risk sets can occur when not all nonabsorbing states are covered, which will result in large variability in the estimates at the corresponding time points. As the number of subjects grows large, this problem is diminished. This problem does not affect transition probabilities out of states which are known to have a sufficient amount of subjects at risk. We therefore suggest to focus more on transition probabilities than cumulative intensities directly.
- When interpreting cumulative intensities, one must realize that the model does not directly estimate the marginal cumulative intensity, but the jumps in the cumulative intensities which are conditional quantities instead.
- Computationally, the multinomial approach is faster than the latent Poisson approach and the time‐homogeneous approach in the presence of exactly observed states. For purely interval‐censored data with constant intensities, the time‐homogeneous approach is best. Additionally, the time‐homogeneous approach allows for the inclusion of covariates.
- The maximal distance between adjacent unique observation times must be sufficiently small for the EM approaches to yield interpretable estimates. As the observation schedule becomes more dense, the variance of the estimators shrinks

In practice, this means that the time‐homogeneous approach is preferred if the intensities are known to be constant and there are no exactly observed states. The multinomial EM approach should be considered for time‐varying intensities and/or in the presence of exactly observed transitions. The Poisson EM approach should only be used if subjects can start in all nonabsorbing states and a theoretical guarantee on consistency of the cumulative intensity is required. When there are large gaps in unique consecutive observation times, the nonparametric models will not recover the underlying intensity well and a model with a smoothness assumption is required.

## 4 Application

In this section, we analyze a part of the Signal‐Tandmobiel study (described in Vanobbergen et al. [22]) using an appropriate multistate model. We are interested in investigating whether estimates obtained after fitting a truly complex multistate model are reasonable.

### 4.1 Signal Tooth Study Data Description

**FIGURE 6:**
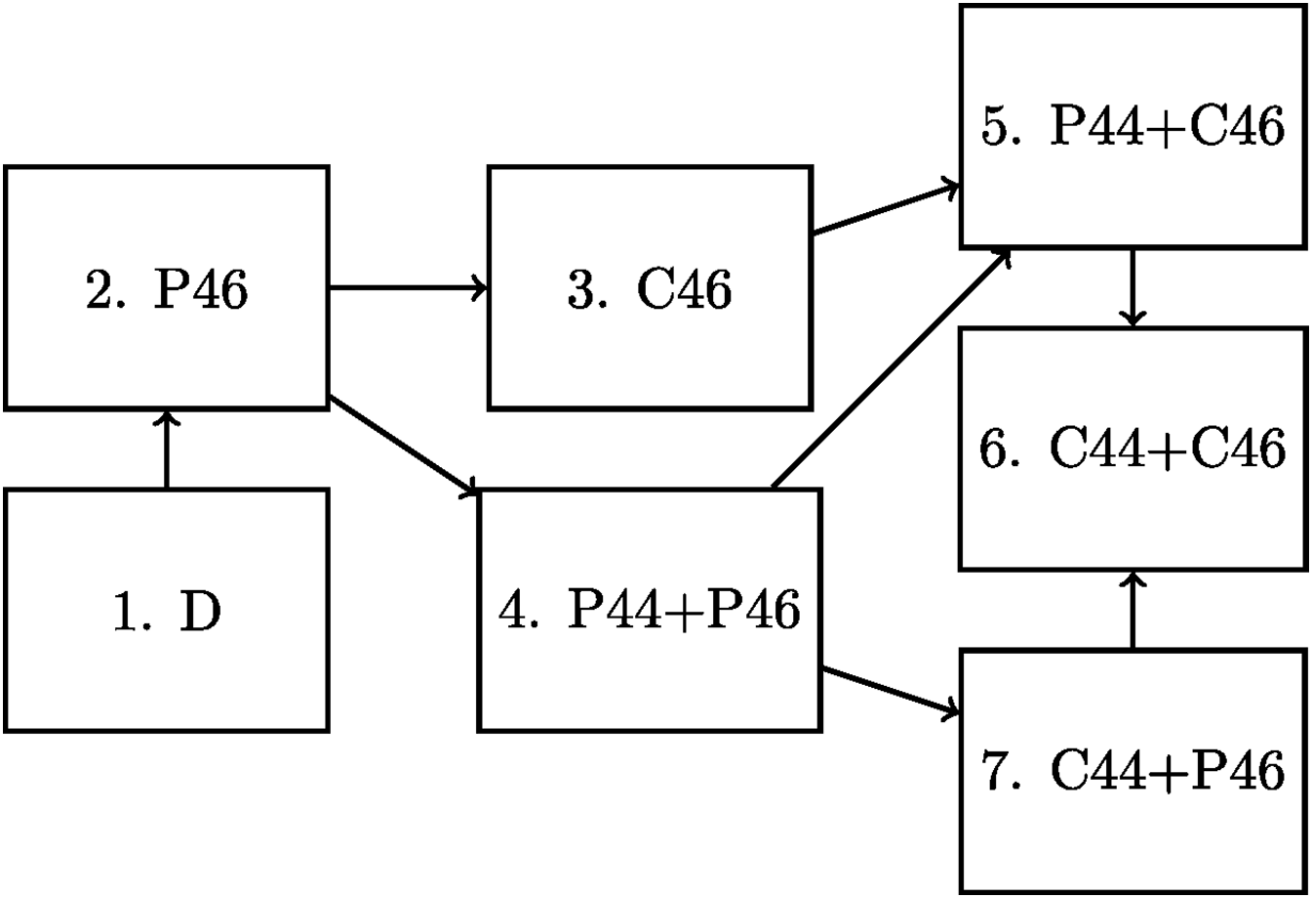
Multistate models used for the analysis of the tooth data set. D stands for deciduous teeth, P for permanent and C for permanent tooth with Caries. The numbers indicate the respective location of the tooth.

The Signal‐Tandmobiel study contains longitudinal information on the occurrence of permanent teeth, caries as well as other (oral) descriptive characteristics between 1996 and 2001 for 4430 children born in 1989. The data set is freely available from the R package icensBKL [1]. Children were examined at most six times per year, with emergence and caries status being determined through inspection by a dentist leading to interval‐censored data. All children start with only deciduous teeth (no permanent teeth). As the interest lies in a nonparametric approach, we leave out any covariate information. Instead, we focus on the emergence and caries status of spatially close permanent teeth 44 and 46. Clearly, a tooth must first emerge before caries can develop. We therefore consider the model shown in Figure 6, with D indicating deciduous teeth only, P a permanent tooth and C a permanent tooth with caries. Only a single child had tooth 44 emerge before tooth 46, therefore this child was removed from the study to significantly simplify the model. Although no formal comparison can be made, we are interested in exploring the dynamics of caries occurrence and the emergence of two neighboring permanent teeth. Additionally, we analyze the rate of caries occurrence on tooth 46, comparing the pathways with and without the intermediary emergence of tooth 44.

For the analysis, we fit the considered model using the multinomial and latent Poisson approach. The EM algorithms were run with initial estimates ${\tilde{\alpha }}_{gh}^{k}=\frac{1}{K}$ if (g,h)∈𝒱 and zero elsewhere until the difference in the estimated intensities was smaller than 0.0001. Additionally, a time‐homogeneous model with piecewise‐constant intensities was also considered, with initial estimates determined by the msm function. Due to numerical issues, only the time‐homogeneous model was simplified to not include states 6 and 7. This simplification should not change the estimates by much, as less than 20 children were observed in both states. As no transitions were observed before 6 years of age, the cut‐points for the homogeneous model were chosen at 6,8 and 10 years to split the relevant time‐frame into three equal parts.

### 4.2 Results

**FIGURE 7:**
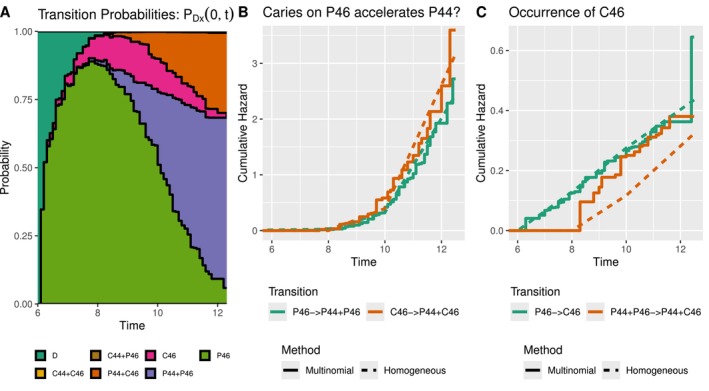
(A) Transition probabilities (multinomial EM) from initial state D over time. The height of a color indicates the probability of being in the corresponding state. (B) Comparison of occurrence of permanent tooth 44 with or without caries on permanent tooth 46. (C) Occurrence of caries on tooth 46 through the two most common pathways.

It took the multinomial algorithm 509 iterations (≈2h) to converge, whereas the Poisson EM algorithm had not converged yet within the first 4000 iterations and was stopped after 18 hours. Although stopped prematurely, the resulting estimates did not differ much between the latent Poisson and multinomial approach. On further inspection, we found that the increase in the likelihood value per iteration was substantially smaller for the latent Poisson approach. Due to the small differences, we only show the results for the multinomial EM approach. Transition probabilities from the initial state to all other states can be seen in Figure 7A. As mentioned before, tooth 46 emerges before 44, with caries on 46 therefore often preceding the emergence of tooth 44. Interestingly, almost none of the children developed caries on both teeth. Additionally, approximately 70 percent of children did not develop caries on any of the 2 considered teeth at the end of the study.

To investigate the dynamics of caries occurrence on tooth 46 and the emergence of tooth 44, we plot the cumulative hazards for the transitions P46→P44+P46 and C46→P44+C46 in Figure 7B. Note that C46 cannot occur before P46, and observing only C46 per definition implies that P44 has not happened yet therefore we expect the C46→P44+C46 transition to have a delayed increase in the cumulative hazard. We can see however that the opposite is true for both models, with the cumulative hazard for the emergence of tooth 44 being larger if caries is first observed on tooth 46, giving rise to the belief that caries is associated with an accelerated occurrence of P44. This is perhaps a bit surprising, as an earlier analysis has shown that occlusal plaque (which usually precedes the occurrence of caries) on tooth 46 has no effect on the emergence of tooth 44 (see Example 4.2 of Bogaerts et al. [1]).

The cumulative hazards of the two most common pathways to caries on tooth 46 are shown in Figure 7C. A similar issue is also present here, as P46 must precede the caries‐free state P44+P46 and therefore we expect the rate of caries occurrence from the second state to increase at a later time point. The overall slopes of the multinomial EM estimates suggest that neither of the two pathways considered is associated with an increased risk of developing caries. The observed increase in the slope for the second transition can be attributed to the smaller risk set at age 8, as a small risk set may lead to large jumps in the estimated cumulative hazard whenever caries is observed. The homogeneous estimates for the two transitions are comparable, with the two transitions displaying similar slopes but shifted in time. Although the same conclusion is reached using the two methods, the homogeneous approach requires the specification of cut‐points. Such a choice requires manual analysis of the data set, in contrast to the EM approaches.

## 5 Discussion

We have derived a nonparametric maximum likelihood estimator for the cumulative transition intensities in multistate models without loops for a combination of interval‐ and right‐censored data. The estimator makes use of the Expectation‐maximization algorithm and is based on the multinomial complete data likelihood for interval‐censored multistate data. We present a necessary and sufficient condition to determine convergence of the algorithm to the true NPMLE. The estimated quantities allow to determine (cumulative) hazards for any transition in the model, as well as transition probabilities over the study period.

In comparison with the latent Poisson EM estimator [20], which was shown to be consistent for the cumulative intensities, our approach yielded almost identical estimates when the initial state can be any nonabsorbing state. Additionally, our estimator yielded interpretable estimates for the transition probabilities when all subjects were initially observed in a single state, contrary to the latent Poisson approach. In such settings, small risk sets can lead to strong variability in the estimates. With larger sample sizes, this problem is mitigated. Finally, the multinomial EM algorithm was found to need significantly less iterations to converge to an estimate, making this approach computationally feasible.

Few approaches existed for the analysis of interval‐censored multistate data until recently, with the time‐homogeneous model being the most popular. In contrast to the time‐homogeneous approach, the nonparametric models allow the underlying cumulative intensities to be modeled with much greater flexibility. Additionally, in the presence of exactly observed (right‐censored) states, our approach is computationally faster than existing time‐homogeneous implementations as it does not rely on numerical optimization techniques.

The multinomial and latent Poisson EM estimators allow for the proper analysis of interval‐censored multistate data, negating the need to use inappropriate or incorrectly specified models. This in turn yields interpretable estimates and allows for unbiased conclusions in the presence of interval‐censoring.

An important consideration when using the proposed estimator is the conditionally independent visit assumption. If future visit times are not independent of the state of the subject since the last visit, the proposed estimator might produce biased estimates. This problem may occur in the commonly used illness‐death model, where sometimes subjects are more likely to visit a doctor shortly after contracting the disease.

The Markov assumption may not always hold. For exactly observed transition times, the methods are available to test the Markov assumption [28] and to estimate state occupation and transition probabilities in the absence of the Markov assumption [29, 30]. For the interval‐censored setting, no tests for the Markov assumption are available yet and alternative methods are lacking as well. Testing the Markov assumption is much more complex in this setting due to the lack of information about actual transition times.

A condition was derived to determine whether the algorithm has converged toward a (local) maximum. An important related problem is determining whether the obtained estimate is the NPMLE. Gentleman and Geyer [31] have provided conditions to check this for the interval‐censored survival setting. Their result can be extended to the multistate setting by considering the KKT conditions (see Appendix Section Interval‐Censored Multistate Data) for the observed‐data likelihood function instead of the expected complete‐data likelihood function (Algorithm 2). This would require the calculation of the derivative of the observed‐likelihood function as well as an expression for the Lagrange multipliers (See Proposition 2). Although the derivative may be approximated numerically, obtaining an expression for the Lagrange multipliers may be very challenging. Another issue that can arise is that the NPMLE might not exist or not be unique [31, 32]. The NPMLE may not be unique when the observed data log‐likelihood function is not strictly concave. For the standard survival setting, a sufficient condition for the uniqueness of the NPMLE is given in Gentleman and Geyer [31]. A similar condition could be derived for interval‐censored multistate data as well.

The proposed estimator yields virtually identical estimates as the latent Poisson approach, thereby implying consistent estimation of the cumulative intensities. A theoretical confirmation of this presumption could be derived following the procedure in Gu et al. [20] Furthermore, the potential extension of our estimator to the semiparametric framework via a proportional hazards approach should be considered. In a semiparametric approach, a hypothesis test of a zero regression coefficient has been used to compare survival curves in an interval‐censored setting [33]. A similar approach could also be used for interval‐censored multistate data, and would allow us to formally compare the cumulative intensities in the Signal‐Tandmobiel study.

Asymptotic properties of the introduced estimator were not discussed in this article. We discuss a few results for the simple two‐state survival model with interval‐censored data (see Chapter 3 of Sun [34]). First of all, the NPMLE of the distribution function is known to have only $n^{1/3}$‐convergence rate and does not have an asymptotic normal distribution. When a certain proportion of failure times is exactly observed (plus some regularity conditions hold) or the support of the distribution function is discrete, the usual $n^{1/2}$‐convergence rate holds, and the resulting asymptotic distribution is normal. Although possible to construct pointwise confidence limits in all of the above situations, either estimation of nontrivial quantities or the asymptotic variance of the distribution is required, which has no closed form. Due to these difficulties, confidence limits in the two‐state setting are usually determined using bootstrap methods due to the low computational cost. Similar results are likely to hold in the multistate setting. Hudgens et al. [32] made the same presumption for the interval‐censored competing risks model: “If the support of the censoring mechanism is discrete and finite, the estimation of the cumulative incidence functions becomes a finite dimensional estimation problem and we expect the NPMLE to have the usual $n^{1/2}$ convergence rate. If the random variables dictating the censoring are treated as continuous, the rate of convergence of the NPMLE will likely not be $n^{1/2}$, and derivation of the limiting distribution will not be trivial.” Part of these presumptions were later reinforced by Gu [21], who has shown the latent Poisson estimator to have a convergence rate of $n^{1/3}$ in the case of continuously distributed censoring. This suggests the use of bootstrap methods to obtain confidence limits, which is unfortunately not feasible due to the computational complexity of the models.

In interval‐censored data, the state of a subject at a visit time is sometimes only known to lie in a set of possible states. It is possible to consider this extension to our model as well, following existing results for the time‐homogeneous model [17]. This would allow us to consider a different right‐censoring mechanism, where the right‐censoring time could also be a time at which the state of a subject is known to not lie in any of the exactly observed states. Furthermore, extensions for truncated data should be considered.

## Conflicts of Interest

The authors declare no conflicts of interest.

## Supporting information




**Data S1.** Supporting Information

## Data Availability

The methods described in this article are implemented in the R package icmstate [35]. The code used to perform the data analysis and simulation study is available on GitHub [36]. The methods described in this article are implemented in the R package icmstate (https://cran.r‐project.org/package=icmstate). The data that support the findings of this study are available in the R package icensBKL (https://cran.r‐project.org/package=icensBKL).

## Appendix A Derivations for the EM Algorithm

### Interval‐Censored Multistate Data

Proposition 1
*Consider the conditional expectation of the complete data log‐likelihood function for interval‐censored multistate data*:

(A1)
$$\begin{array}{ll} & 𝔼[\ell^{C}\;|\;O,\tilde{\alpha }]=\overset{K}{\underset{k=1}{\sum }}\underset{(g,h)\in 𝒱}{\sum }\left\{𝔼\left[d_{gh}^{k}\;|\;O,\tilde{\alpha }\right]\log(\alpha_{gh}^{k})-\alpha_{gh}^{k}𝔼\left[Y_{g}^{k}\;|\;O,\tilde{\alpha }\right]\right\} \end{array}$$

*The following conditional expectations when no transition times are known exactly are given by*:

$$\begin{array}{ll} d_{gh}^{k}(\tilde{\alpha }):=𝔼\left[d_{gh}^{k}\;|\;O,\tilde{\alpha }\right] & =\overset{n}{\underset{i=1}{\sum }}\frac{{\tilde{P}}_{a_{i}^{k},g}(l_{i}^{k},\tau_{k-1})\cdot {\tilde{\alpha }}_{gh}^{k}\cdot {\tilde{P}}_{h,b_{i}^{k}}(\tau_{k},r_{i}^{k})}{{\tilde{P}}_{a_{i}^{k},b_{i}^{k}}(l_{i}^{k},r_{i}^{k})},\\ Y_{g}^{k}(\tilde{\alpha }):=𝔼\left[Y_{g}^{k}\;|\;O,\tilde{\alpha }\right] & =\overset{n}{\underset{i=1}{\sum }}\frac{{\tilde{P}}_{a_{i}^{k},g}(l_{i}^{k},\tau_{k-1})\cdot {\tilde{P}}_{g,b_{i}^{k}(\tau_{k}-,r_{i}^{k})}}{{\tilde{P}}_{a_{i}^{k},b_{i}^{k}}(l_{i}^{k},r_{i}^{k})} \end{array}$$

*with*  *the product integral of the current estimates of the cumulative transition intensities*.

For ease of notation, we define $\tilde{𝔼}[\cdot ]=𝔼[\cdot \;|\;\tilde{\alpha }]$ and $\tilde{\mathbb{P}}[\cdot ]=\mathbb{P}[\cdot \;|\;\tilde{\alpha }]$ as the conditional expectation and probability given the current estimates of the cumulative transition intensities. As we have assumed that the cumulative intensity is a right‐continuous step function with jumps only in 𝒯, we have:

$$\begin{array}{ll} 𝔼[d_{gh}^{k}\;|\;O,\tilde{\alpha }] & =\overset{n}{\underset{i=1}{\sum }}𝔼[1\{X_{i}(\tau_{k})=h,X_{i}(\tau_{k}-)=g\}\;|\;O,\tilde{\alpha }]\\ & =\overset{n}{\underset{i=1}{\sum }}\mathbb{P}(X_{i}(\tau_{k})=h,X_{i}(\tau_{k}-)=g\;|\;{𝕏}_{i}^{O},\tilde{\alpha })\\ & =:\overset{n}{\underset{i=1}{\sum }}d_{gh,i}^{k}(\tilde{\alpha })=:d_{gh}^{k}(\tilde{\alpha })\\ 𝔼\left[Y_{g}^{k}\;|\;O,\tilde{\alpha }\right] & =\overset{n}{\underset{i=1}{\sum }}𝔼[1\{X_{i}(\tau_{k}-)=g\}\;|\;O,\tilde{\alpha }]\\ & =\overset{n}{\underset{i=1}{\sum }}\mathbb{P}(X_{i}(\tau_{k}-)=g\;|\;{𝕏}_{i}^{O},\tilde{\alpha })\\ & =:\overset{n}{\underset{n=1}{\sum }}Y_{g,i}^{k}(\tilde{\alpha })=:Y_{g}^{k}(\tilde{\alpha }) \end{array}$$

with $\tau_{k}-$ the time just before $\tau_{k}$ and ${𝕏}_{i}^{O}$ the observed information for subject i as in Section 2.2. Fix some k∈{1,…,K} and i∈{1,…,n}. Define ${\tilde{F}}_{i}^{k}=F_{i}^{k}\setminus \{X_{i}(r_{i}^{k})\}$ and ${\tilde{B}}_{i}^{k}=B_{i}^{k}\setminus \{X_{i}(l_{i}^{k})\}$. We look at the individual terms separately:

(A2)
$$\begin{array}{ll} Y_{g,i}^{k}(\tilde{\alpha }) & =\tilde{\mathbb{P}}(X_{i}(\tau_{k}-)=g\;|\;{𝕏}_{i}^{O})\\ & =\tilde{\mathbb{P}}(X_{i}(\tau_{k}-)=g\;|\;{\tilde{B}}_{i}^{k},X_{i}(l_{i}^{k})=a_{i}^{k},X_{i}(r_{i}^{k})=b_{i}^{k},{\tilde{F}}_{i}^{k})\\ & =\frac{\tilde{\mathbb{P}}(X_{i}(r_{i}^{k})=b_{i}^{k},{\tilde{F}}_{i}^{k}\;|\;X_{i}(\tau_{k}-)=g,{\tilde{B}}_{i}^{k},X_{i}(l_{i}^{k})=a_{i}^{k})\cdot \tilde{\mathbb{P}}(X_{i}(\tau_{k}-)=g\;|\;{\tilde{B}}_{i}^{k},X_{i}(l_{i}^{k})=a_{i}^{k})}{\tilde{\mathbb{P}}(X_{i}(r_{i}^{k})=b_{i}^{k},{\tilde{F}}_{i}^{k}\;|\;X_{i}(l_{i}^{k})=a_{i}^{k},{\tilde{B}}_{i}^{k})}\\ & \overset{Markov}{=}\frac{\tilde{\mathbb{P}}(X_{i}(r_{i}^{k})=b_{i}^{k},{\tilde{F}}_{i}^{k}\;|\;X_{i}(\tau_{k}-)=g)\cdot \tilde{\mathbb{P}}(X_{i}(\tau_{k}-)=g\;|\;X_{i}(l_{i}^{k})=a_{i}^{k})}{\tilde{\mathbb{P}}(X_{i}(r_{i}^{k})=b_{i}^{k},{\tilde{F}}_{i}^{k}\;|\;X_{i}(l_{i}^{k})=a_{i}^{k})}\\ & =\frac{\tilde{\mathbb{P}}({\tilde{F}}_{i}^{k}\;|\;X_{i}(r_{i}^{k})=b_{i}^{k},X_{i}(\tau_{k}-)=g)\cdot \tilde{\mathbb{P}}(X_{i}(r_{i}^{k})=b_{i}^{k}\;|\;X_{i}(\tau_{k}-)=g)\cdot \tilde{\mathbb{P}}(X_{i}(\tau_{k}-)=g\;|\;X_{i}(l_{i}^{k})=a_{i}^{k})}{\tilde{\mathbb{P}}({\tilde{F}}_{i}^{k}\;|\;X_{i}(r_{i}^{k})=b_{i}^{k},X_{i}(l_{i}^{k})=a_{i}^{k})\cdot \tilde{\mathbb{P}}(X_{i}(r_{i}^{k})=b_{i}^{k}\;|\;X_{i}(l_{i}^{k})=a_{i}^{k})}\\ & \overset{Markov}{=}\frac{\tilde{\mathbb{P}}(X_{i}(r_{i}^{k})=b_{i}^{k}\;|\;X_{i}(\tau_{k}-)=g)\cdot \tilde{\mathbb{P}}(X_{i}(\tau_{k}-)=g\;|\;X_{i}(l_{i}^{k})=a_{i}^{k})}{\tilde{\mathbb{P}}(X_{i}(r_{i}^{k})=b_{i}^{k}\;|\;X_{i}(l_{i}^{k})=a_{i}^{k})}\\ & =\frac{{\tilde{P}}_{a_{i}^{k},g}(l_{i}^{k},\tau_{k}-)\cdot {\tilde{P}}_{g,b_{i}^{k}}(\tau_{k}-,r_{i}^{k})}{{\tilde{P}}_{a_{i}^{k},b_{i}^{k}}(l_{i}^{k},r_{i}^{k})} \end{array}$$

with ${\tilde{P}}_{gh}(s,t)$ the (g,h)th entry of the product integral over the current estimates . We continue to the next term:

(A3)
$$\begin{array}{ll} d_{gh,i}^{k} & =\tilde{\mathbb{P}}(X_{i}(\tau_{k}-)=g,X_{i}(\tau_{k})=h\;|\;{𝕏}_{i}^{O})\\ & =\tilde{\mathbb{P}}(X_{i}(\tau_{k}-)=g\;|\;{𝕏}_{i}^{O})\cdot \tilde{\mathbb{P}}(X_{i}(\tau_{k})=h\;|\;X_{i}(\tau_{k}-)=g,{𝕏}_{i}^{O}) \end{array}$$

The first term is simply $Y_{g,i}^{k}(\tilde{\alpha })$, so we take a further look at the second term:

(A4)
$$\begin{array}{ll} & \tilde{\mathbb{P}}(X_{i}(\tau_{k})=h\;|\;X_{i}(\tau_{k}-)=g,{𝕏}_{i}^{O})\\ & \;=\frac{\tilde{\mathbb{P}}(X_{i}(\tau_{k})=h,X_{i}(\tau_{k}-)=g,B_{i}^{k},F_{i}^{k})}{\tilde{\mathbb{P}}(X_{i}(\tau_{k}-)=g,B_{i}^{k},F_{i}^{k})}\\ & \;=\frac{\tilde{\mathbb{P}}(F_{i}^{k}\;|\;X_{i}(\tau_{k})=h)\cdot \tilde{\mathbb{P}}(X_{i}(\tau_{k})=h\;|\;X_{i}(\tau_{k}-)=g)}{\tilde{\mathbb{P}}(F_{i}^{k}\;|\;X_{i}(\tau_{k}-)=g)}\\ & \;=\frac{\tilde{\mathbb{P}}(X_{i}(r_{i}^{k})=b_{i}^{k}\;|\;X_{i}(\tau_{k})=h)\cdot {\tilde{\alpha }}_{gh}^{k}}{\tilde{\mathbb{P}}(X_{i}(r_{i}^{k})=b_{i}^{k}\;|\;X_{i}(\tau_{k}-)=g)}\\ & \;=\frac{{\tilde{P}}_{h,b_{i}^{k}}(\tau_{k},r_{i}^{k})\cdot {\tilde{\alpha }}_{gh}^{k}}{{\tilde{P}}_{g,b_{i}^{k}}(\tau_{k}-,r_{i}^{k})} \end{array}$$

As the cumulative intensities were assumed to be right‐continuous step functions, we obtain that:

$$\begin{array}{l} {\tilde{P}}_{gh}(\tau_{k}-,t)={\tilde{P}}_{gh}(\tau_{k-1},t) \end{array}$$

for any k∈1,…,K and any $t>\tau_{k}$. Using this fact and substituting Equations (A4) and (A2) into Equation (A3) and summing over n we obtain the final result.

Proposition 2
*The conditional expected value of the complete data log‐likelihood for interval‐censored multistate data*(*see Equation* (*A1*)) *over the optimization region*:

$$\begin{array}{ll} C_{\alpha } & =\left\{\alpha_{gh}^{k},g\neq h\in ℋ,k=1,\ldots ,K;\alpha_{gh}^{k}\geq 0,\overset{H}{\underset{h\neq g=1}{\sum }}\alpha_{gh}^{k}\leq 1\right\} \end{array}$$

*is maximised by the following expression*:

$$\begin{array}{ll} \alpha_{gh}^{k} & =\left\{\begin{array}{ll} \frac{d_{gh}^{k}(\tilde{\alpha })}{Y_{g}^{k}(\tilde{\alpha })},\; & \mu_{g}^{k}=0,\\ \frac{d_{gh}^{k}(\tilde{\alpha })}{\sum_{h\leftarrow g}d_{gh}^{k}(\tilde{\alpha })},\; & \mu_{g}^{k}>0 \end{array}\right. \end{array}$$

*with* $\mu_{g}^{k}=\max\left(0,\sum_{h\leftarrow g}d_{gh}^{k}(\tilde{\alpha })-Y_{g}^{k}(\tilde{\alpha })\right).$

Maximization of the complete data log‐likelihood function can be phrased as a convex optimization problem. The primal problem is given by (denoting $l^{C}(\alpha )=\tilde{𝔼}[\ell^{C}]$):

$$\begin{array}{ll} \max & \;l^{C}(\alpha )=\overset{K}{\underset{k=1}{\sum }}\underset{(g,h)\in 𝒱}{\sum }d_{gh}^{k}(\tilde{\alpha })\log(\alpha_{gh}^{k})-\overset{K}{\underset{k=1}{\sum }}\underset{(g,h)\in 𝒱}{\sum }\alpha_{gh}^{k}Y_{g}^{k}(\tilde{\alpha })\\ \text{s.t.}\; & \;\alpha_{gh}^{k}\geq 0,\;g\neq h\in ℋ,k\in \{1,\ldots ,K\},\\ & \;\underset{h\neq g}{\sum }\alpha_{gh}^{k}\leq 1,\;g\in ℋ,k\in \{1,\ldots ,K\} \end{array}$$

or rewritten as a minimization problem:

$$\begin{array}{ll} \min & \;-l^{C}(\alpha )=\overset{K}{\underset{k=1}{\sum }}\underset{(g,h)\in 𝒱}{\sum }\alpha_{gh}^{k}Y_{g}^{k}(\tilde{\alpha })-\overset{K}{\underset{k=1}{\sum }}\underset{(g,h)\in 𝒱}{\sum }d_{gh}^{k}(\tilde{\alpha })\log(\alpha_{gh}^{k})\\ \text{s.t.}\; & \;-\alpha_{gh}^{k}\leq 0,\;g\neq h\in ℋ,k\in \{1,\ldots ,K\},\\ & \;\underset{h\neq g}{\sum }\alpha_{gh}^{k}-1\leq 0,\;g\in ℋ,k\in \{1,\ldots ,K\} \end{array}$$

As the logarithm is a concave function, minus the logarithm is convex and the addition of linear terms results in a convex function $-l^{C}(\alpha )$. The Lagrangian is given by:

$$\begin{array}{ll} ℒ(\alpha ,\mu ,\lambda ) & =\overset{K}{\underset{k=1}{\sum }}\underset{(g,h)\in 𝒱}{\sum }\alpha_{gh}^{k}Y_{g}^{k}(\tilde{\alpha })-\overset{K}{\underset{k=1}{\sum }}\underset{(g,h)\in 𝒱}{\sum }d_{gh}^{k}(\tilde{\alpha })\log(\alpha_{gh}^{k})\\ & -\overset{H}{\underset{g=1}{\sum }}\overset{H}{\underset{h\neq g}{\sum }}\overset{K}{\underset{k=1}{\sum }}\lambda_{gh}^{k}\alpha_{gh}^{k}+\overset{H}{\underset{g=1}{\sum }}\overset{K}{\underset{k=1}{\sum }}\mu_{g}^{k}\left(\overset{H}{\underset{h\neq g=1}{\sum }}\alpha_{gh}^{k}-1\right) \end{array}$$

with $\lambda_{gh}^{k},\mu_{g}^{k}$ called Lagrange multipliers. A necessary and sufficient condition for α to be a (local) minimum to above minimization problem is given by the KarushâĂŤKuhn–Tucker (KKT) conditions (see Section 5.5.3 of [25]):

$$\begin{array}{ll} & (1)\;\frac{\partial ℒ(\alpha ,\mu )}{\partial \alpha_{gh}^{k}}=Y_{g}^{k}-\frac{d_{gh}^{k}}{\alpha_{gh}^{k}}-\lambda_{gh}^{k}+\mu_{g}^{k}=0,g\neq h\in ℋ,k\in \{1,\ldots ,K\},\\ & (2)\;\lambda_{gh}^{k}\alpha_{gh}^{k}=0,\;g\neq h\in ℋ,k\in \{1,\ldots ,K\},\\ & (3)\;\;\mu_{g}^{k}\left(\underset{h\leftarrow g}{\sum }\alpha_{gh}^{k}-1\right)=0,\;g\in ℋ,k\in \{1,\ldots ,K\},\\ & (4)\;\alpha_{gh}^{k}\geq 0,\;g\neq h\in ℋ,k\in \{1,\ldots ,K\},\\ & (5)\;\underset{h\leftarrow g}{\sum }\alpha_{gh}^{k}-1\leq 0,\;g\in ℋ,k\in \{1,\ldots ,K\},\\ & \left(6)\;\lambda_{gh}^{k},\mu_{g}^{k}\geq 0,\;g\neq h\in ℋ,k\in \{1,\ldots ,K\right\} \end{array}$$

When $\sum_{h\neq g=1}^{H}\alpha_{gh}^{k}-1<0$, constraint (3) is called inactive. When the constraint is inactive, we must have that $\mu_{g}^{k}=0$. When the constraint is active, $\mu_{g}^{k}$ tells us how much we can improve the objective function by changing the right‐hand side of the inequality constraint. Note that $\mu_{g}^{k}$ must be positive, as increasing the right hand side of inequality constraint (5) can improve the objective function. From equality (1) in the KKT conditions, we recover:

$$\begin{array}{l} Y_{g}^{k}(\tilde{\alpha })-\frac{d_{gh}^{k}(\tilde{\alpha })}{\alpha_{gh}^{k}}-\lambda_{gh}^{k}+\mu_{g}^{k}=0 \end{array}$$

Note that this is undefined for $\alpha_{gh}^{k}=0$. Clearly, if $d_{gh}^{k}(\tilde{\alpha })=0$, then such a transition is not possible under our current estimate of α, so we define $\frac{0}{0}=0$. Suppose that $\alpha_{gh}^{k}>0$. In that case, the complementary slackness condition $\lambda_{gh}^{k}\alpha_{gh}^{k}=0$ ensures that $\lambda_{gh}^{k}=0$. We obtain:

(A5)
$$\begin{array}{l} Y_{g}^{k}(\tilde{\alpha })-\frac{d_{gh}^{k}(\tilde{\alpha })}{\alpha_{gh}^{k}}+\mu_{g}^{k}=0 \end{array}$$

Multiply both sides by $\alpha_{gh}^{k}>0$:

$$\begin{array}{l} \alpha_{gh}^{k}Y_{g}^{k}(\tilde{\alpha })-d_{gh}^{k}(\tilde{\alpha })+\alpha_{gh}^{k}\mu_{g}^{k}=0 \end{array}$$

Sum over all states h directly connected to g:

$$\begin{array}{l} Y_{g}^{k}(\tilde{\alpha })\underset{h\leftarrow g}{\sum }\alpha_{gh}^{k}-\underset{h\leftarrow g}{\sum }d_{gh}^{k}(\tilde{\alpha })+\mu_{g}^{k}\underset{h\leftarrow g}{\sum }\alpha_{gh}^{k}=0 \end{array}$$

We add and subtract $\mu_{g}^{k}$ to obtain:

$$\begin{array}{l} Y_{g}^{k}(\tilde{\alpha })\underset{h\leftarrow g}{\sum }\alpha_{gh}^{k}-\underset{h\leftarrow g}{\sum }d_{gh}^{k}(\tilde{\alpha })+\mu_{g}^{k}\left(\underset{h\leftarrow g}{\sum }\alpha_{gh}^{k}-1\right)+\mu_{g}^{k}=0 \end{array}$$

and using KKT Condition (3):

$$\begin{array}{l} \mu_{g}^{k}=\underset{h\leftarrow g}{\sum }d_{gh}^{k}(\tilde{\alpha })-Y_{g}^{k}(\tilde{\alpha })\underset{h\leftarrow g}{\sum }\alpha_{gh}^{k} \end{array}$$

Finally, we obtain:

(A6)
$$\begin{array}{l} \underset{h\leftarrow g}{\sum }\alpha_{gh}^{k}=\frac{-\mu_{g}^{k}+\sum_{h\leftarrow g}d_{gh}^{k}(\tilde{\alpha })}{Y_{g}^{k}(\tilde{\alpha })} \end{array}$$

Alternatively, we rewrite Equation (A5) to obtain:

(A7)
$$\begin{array}{l} \alpha_{gh}^{k}=\frac{d_{gh}^{k}(\tilde{\alpha })}{Y_{g}^{k}(\tilde{\alpha })+\mu_{g}^{k}} \end{array}$$

and summing over h←g:

(A8)
$$\begin{array}{l} \underset{h\leftarrow g}{\sum }\alpha_{gh}^{k}=\frac{\sum_{h\leftarrow g}d_{gh}^{k}(\tilde{\alpha })}{Y_{g}^{k}(\tilde{\alpha })+\mu_{g}^{k}} \end{array}$$

Now equating Equations (A6) and (A8) we obtain:

$$\begin{array}{l} \frac{-\mu_{g}^{k}+\sum_{h\leftarrow g}d_{gh}^{k}(\tilde{\alpha })}{Y_{g}^{k}(\tilde{\alpha })}=\frac{\sum_{h\leftarrow g}d_{gh}^{k}(\tilde{\alpha })}{Y_{g}^{k}(\tilde{\alpha })+\mu_{g}^{k}} \end{array}$$

After some algebra we obtain:

$$\begin{array}{l} \mu_{g}^{k}\left(\mu_{g}^{k}+Y_{g}^{k}(\tilde{\alpha })-\underset{h\leftarrow g}{\sum }d_{gh}^{k}(\tilde{\alpha })\right)=0 \end{array}$$

Taking into account that $\mu_{g}^{k}\geq 0$ for a feasible solution, we must have:

(A9)
$$\begin{array}{l} \mu_{g}^{k}=\max\left(0,\underset{h\leftarrow g}{\sum }\;\overset{k}{\underset{gh}{d}}(\tilde{\alpha })-\overset{k}{\underset{g}{Y}}(\tilde{\alpha })\right) \end{array}$$

Substituting Equation (A9) into Equation (A7) we obtain the final result:

(A10)
$$\begin{array}{ll} \alpha_{gh}^{k} & =\left\{\begin{array}{ll} \frac{d_{gh}^{k}(\tilde{\alpha })}{Y_{g}^{k}(\tilde{\alpha })},\; & \mu_{g}^{k}=0,\\ \frac{d_{gh}^{k}(\tilde{\alpha })}{\sum_{h\leftarrow g}d_{gh}^{k}(\tilde{\alpha })},\; & \mu_{g}^{k}>0 \end{array}\right. \end{array}$$

### Interval Censored Multistate Data With Exactly Observed States

Proposition 3
*Consider the conditional expectation of the complete data log‐likelihood function for interval‐censored multistate data*:

(A11)
$$\begin{array}{ll} & 𝔼[\ell^{C}\;|\;O,\tilde{\alpha }]=\overset{K}{\underset{k=1}{\sum }}\underset{(g,h)\in 𝒱}{\sum }\left\{𝔼\left[d_{gh}^{k}\;|\;O,\tilde{\alpha }\right]\log(\alpha_{gh}^{k})-\alpha_{gh}^{k}𝔼\left[Y_{g}^{k}\;|\;O,\tilde{\alpha }\right]\right\} \end{array}$$

*The conditional expectations for interval‐censored multistate data with transition times exactly known for a subset of states* ℰ⊆ℋ *are given by*:

$$\begin{array}{ll} d_{gh,i}^{k}(\tilde{\alpha }) & :=𝔼\left[d_{gh,i}^{k}\;|\;O,\tilde{\alpha }\right]\\ & =\left\{\begin{array}{ll} \frac{{\tilde{P}}_{a_{i}^{k},g}(l_{i}^{k},\tau_{k-1})\cdot {\tilde{\alpha }}_{gh}^{k}\cdot {\tilde{P}}_{h,b_{i}^{k}}(\tau_{k},r_{i}^{k})}{{\tilde{P}}_{a_{i}^{k},b_{i}^{k}}(l_{i}^{k},r_{i}^{k})},\; & \text{if}\;b_{i}^{k}\notin ℰ\\ \frac{{\tilde{P}}_{a_{i}^{k}g}(l_{i}^{k},\tau_{k-1})\cdot {\tilde{\alpha }}_{gh}^{k}\cdot \sum_{m\in {ℛ}_{b_{i}^{k}}}{\tilde{\alpha }}_{mb_{i}^{k}}^{k_{r_{i}}}{\tilde{P}}_{hm}(\tau_{k},\tau_{k_{r_{i}}-1})}{\sum_{m\in {ℛ}_{b_{i}^{k}}}{\tilde{\alpha }}_{mb_{i}^{k}}^{k_{r_{i}}}{\tilde{P}}_{a_{i}^{k}m}(l_{i}^{k},\tau_{k_{r_{i}}-1})},\; & \text{if}\;b_{i}^{k}\in ℰ \end{array}\right.\\ Y_{g,i}^{k}(\tilde{\alpha }) & :=𝔼\left[Y_{g,i}^{k}\;|\;O,\tilde{\alpha }\right]\\ & =\left\{\begin{array}{ll} \frac{{\tilde{P}}_{a_{i}^{k},g}(l_{i}^{k},\tau_{k-1})\cdot {\tilde{P}}_{g,b_{i}^{k}}(\tau_{k-1},r_{i}^{k})}{{\tilde{P}}_{a_{i}^{k},b_{i}^{k}}(l_{i}^{k},r_{i}^{k})},\; & \text{if}\;b_{i}^{k}\notin ℰ\\ \frac{{\tilde{P}}_{a_{i}^{k}g}(l_{i}^{k},\tau_{k-1})\cdot \sum_{m\in {ℛ}_{b_{i}^{k}}}{\tilde{\alpha }}_{mb_{i}^{k}}^{k_{r_{i}}}{\tilde{P}}_{gm}(\tau_{k-1},\tau_{k_{r_{i}}-1})}{\sum_{m\in {ℛ}_{b_{i}^{k}}}{\tilde{\alpha }}_{mb_{i}^{k}}^{k_{r_{i}}}{\tilde{P}}_{a_{i}^{k}m}(l_{i}^{k},\tau_{k_{r_{i}}-1})},\; & \text{if}\;b_{i}^{k}\in ℰ \end{array}\right. \end{array}$$

*with* $\tau_{k_{r_{i}}}:=r_{i}^{k}$, $d_{gh}^{k}(\tilde{\alpha })=\sum_{i=1}^{n}d_{gh,i}^{k}(\tilde{\alpha })$, $Y_{g}^{k}(\tilde{\alpha })=\sum_{i=1}^{n}Y_{g,i}^{k}(\tilde{\alpha })$ *and*  *the product integral of the current estimates of the cumulative transition intensities*.

We follow the beginning of the proof of Proposition 1. Fix k and i and consider the situation when $b_{i}^{k}\notin ℰ$. It is apparent that the result from Proposition 1 still holds, as no additional information is available in that case. We therefore consider the case when $b_{i}^{k}\in ℰ$ and look at the individual terms separately:

$$\begin{array}{ll} Y_{g,i}^{k}(\tilde{\alpha }) & =\tilde{\mathbb{P}}(X_{i}(\tau_{k}-)=g\;|\;{𝕏}_{i}^{O})\\ & =\tilde{\mathbb{P}}(X_{i}(\tau_{k}-)=g\;|\;X_{i}(r_{i}^{k})=b_{i}^{k},{\tilde{B}}_{i}^{k},{\tilde{F}}_{i}^{k},X_{i}(l_{i}^{k})=a_{i}^{k},b_{i}^{k}\in ℰ)\\ & =\frac{\tilde{\mathbb{P}}({\tilde{F}}^{k},X_{i}(r_{i}^{k})=b_{i}^{k}\;|\;X_{i}(\tau_{k}-)=g,{\tilde{B}}_{i}^{k},X_{i}(l_{i}^{k})=a_{i}^{k},b_{i}^{k}\in ℰ)\cdot \tilde{\mathbb{P}}(X_{i}(\tau_{k}-)=g\;|\;X_{i}(l_{i}^{k})=a_{i}^{k},{\tilde{B}}_{i}^{k},b_{i}^{k}\in ℰ)}{\tilde{\mathbb{P}}({\tilde{F}}^{k},X_{i}(r_{i}^{k})=b_{i}^{k}\;|\;X_{i}(l_{i}^{k})=a_{i}^{k},{\tilde{B}}_{i}^{k},b_{i}^{k}\in ℰ)}\\ & \overset{Markov}{=}\frac{\tilde{\mathbb{P}}({\tilde{F}}^{k},X_{i}(r_{i}^{k})=b_{i}^{k}\;|\;X_{i}(\tau_{k}-)=g,b_{i}^{k}\in ℰ)\cdot \tilde{\mathbb{P}}(X_{i}(\tau_{k}-)=g\;|\;X_{i}(l_{i}^{k})=a_{i}^{k},b_{i}^{k}\in ℰ)}{\tilde{\mathbb{P}}({\tilde{F}}^{k},X_{i}(r_{i}^{k})=b_{i}^{k}\;|\;X_{i}(l_{i}^{k})=a_{i}^{k},b_{i}^{k}\in ℰ)}\\ & =\frac{\tilde{\mathbb{P}}({\tilde{F}}^{k},X_{i}(r_{i}^{k})=b_{i}^{k}\;|\;X_{i}(\tau_{k}-)=g,b_{i}^{k}\in ℰ)\cdot \tilde{\mathbb{P}}(X_{i}(\tau_{k}-)=g\;|\;X_{i}(l_{i}^{k})=a_{i}^{k})}{\tilde{\mathbb{P}}({\tilde{F}}^{k},X_{i}(r_{i}^{k})=b_{i}^{k}\;|\;X_{i}(l_{i}^{k})=a_{i}^{k},b_{i}^{k}\in ℰ)}\\ & =\frac{\tilde{\mathbb{P}}(X_{i}(r_{i}^{k})=b_{i}^{k}\;|\;X_{i}(\tau_{k}-)=g,b_{i}^{k}\in ℰ)\cdot \tilde{\mathbb{P}}(X_{i}(\tau_{k}-)=g\;|\;X_{i}(l_{i}^{k})=a_{i}^{k})}{\tilde{\mathbb{P}}(X_{i}(r_{i}^{k})=b_{i}^{k}\;|\;X_{i}(l_{i}^{k})=a_{i}^{k},b_{i}^{k}\in ℰ)} \end{array}$$

${𝕏}_{i}^{O}$ is the observed information for subject i as defined in Section 2.2. As $b_{i}^{k}\in ℰ$, the subject must have been in ${ℛ}_{b_{i}^{k}}$ at time $r_{i}^{k}-$. We therefore define $k_{r_{i}}$ such that $\tau_{k_{r_{i}}}=r_{i}^{k}$ and condition on time $\tau_{k_{r_{i}}-1}$ using the law of total probability in the numerator above:

$$\begin{array}{ll} & \tilde{\mathbb{P}}(X_{i}(r_{i}^{k})=b_{i}^{k}\;|\;X_{i}(\tau_{k}-)=g,b_{i}^{k}\in ℰ)\\ & \;=\underset{m\in {ℛ}_{b_{i}^{k}}}{\sum }\tilde{\mathbb{P}}(X_{i}(r_{i}^{k})=b_{i}^{k}\;|\;X_{i}(\tau_{k}-)=g,X_{i}(r_{i}^{k}-)=m,b_{i}^{k}\in ℰ)\cdot \\ & \tilde{\mathbb{P}}(X_{i}(r_{i}^{k}-)=m\;|\;X_{i}(\tau_{k}-)=g,b_{i}^{k}\in ℰ)\\ & \;=\underset{m\in {ℛ}_{b_{i}^{k}}}{\sum }\tilde{\mathbb{P}}(X_{i}(r_{i}^{k})=b_{i}^{k}\;|\;X_{i}(r_{i}^{k}-)=m,b_{i}^{k}\in ℰ)\cdot \\ & \tilde{\mathbb{P}}(X_{i}(r_{i}^{k}-)=m\;|\;X_{i}(\tau_{k}-)=g)\\ & \;=\underset{m\in {ℛ}_{b_{i}^{k}}}{\sum }{\tilde{P}}_{mb_{i}^{k}}(r_{i}^{k}-,r_{i}^{k}){\tilde{P}}_{gm}(\tau_{k-1},r_{i}^{k}-)\\ & \;=\underset{m\in {ℛ}_{b_{i}^{k}}}{\sum }{\tilde{\alpha }}_{mb_{i}^{k}}^{k_{r_{i}}}{\tilde{P}}_{gm}(\tau_{k-1},\tau_{k_{r_{i}}-1}) \end{array}$$

Similarly, we obtain for the denominator:

$$\begin{array}{ll} \tilde{\mathbb{P}}(X_{i}(r_{i}^{k}) & =b_{i}^{k}\;|\;X_{i}(l_{i}^{k})=a_{i}^{k},b_{i}^{k}\in ℰ)=\underset{m\in {ℛ}_{b_{i}^{k}}}{\sum }{\tilde{\alpha }}_{mb_{i}^{k}}^{k_{r_{i}}}{\tilde{P}}_{a_{i}^{k}m}(l_{i}^{k},\tau_{k_{r_{i}}-1}) \end{array}$$

Substituting these back into the original expression we obtain:

(A12)
$$\begin{array}{ll} Y_{g,i}^{k}(\tilde{\alpha }) & =\frac{{\tilde{P}}_{a_{i}^{k}g}(l_{i}^{k},\tau_{k-1})\cdot \sum_{m\in {ℛ}_{b_{i}^{k}}}{\tilde{\alpha }}_{mb_{i}^{k}}^{k_{r_{i}}}{\tilde{P}}_{gm}(\tau_{k-1},\tau_{k_{r_{i}}-1})}{\sum_{m\in {ℛ}_{b_{i}^{k}}}{\tilde{\alpha }}_{mb_{i}^{k}}^{k_{r_{i}}}{\tilde{P}}_{a_{i}^{k}m}(l_{i}^{k},\tau_{k_{r_{i}}-1})} \end{array}$$

We continue on to:

$$\begin{array}{ll} & d_{gh,i}^{k}(\tilde{\alpha })=\tilde{\mathbb{P}}(X_{i}(\tau_{k}-)=g,X_{i}(\tau_{k})=h\;|\;{𝕏}_{i}^{O})\\ & \;=\tilde{\mathbb{P}}(X_{i}(\tau_{k}-)=g\;|\;{𝕏}_{i}^{O})\cdot \tilde{\mathbb{P}}(X_{i}(\tau_{k})=h\;|\;X_{i}(\tau_{k}-)=g,{𝕏}_{i}^{O}) \end{array}$$

and again focus only on the second term:

$$\begin{array}{ll} & \tilde{\mathbb{P}}(X_{i}(\tau_{k})=h\;|\;X_{i}(\tau_{k}-)=g,{𝕏}_{i}^{O})\\ & =\frac{\begin{array}{l} \tilde{\mathbb{P}}(F^{k}\;|\;X_{i}(\tau_{k})=h,b_{i}^{k}\in ℰ)\cdot \tilde{\mathbb{P}}(X_{i}(\tau_{k})=h\;|\;X_{i}(\tau_{k}-)=g,b_{i}^{k}\in ℰ) \end{array}}{\tilde{\mathbb{P}}(F^{k}\;|\;X_{i}(\tau_{k}-)=g,b_{i}^{k}\in ℰ)}\\ & =\frac{\tilde{\mathbb{P}}(X_{i}(r_{i}^{k})=b_{i}^{k}\;|\;X_{i}(\tau_{k})=h,b_{i}^{k}\in ℰ)\cdot {\tilde{\alpha }}_{gh}^{k}}{\tilde{\mathbb{P}}(X_{i}(r_{i}^{k})=b_{i}^{k}\;|\;X_{i}(\tau_{k}-)=g,b_{i}^{k}\in ℰ)} \end{array}$$

The term in the denominator was already expanded above, so we focus on the term in the numerator (in a similar way as before):

$$\begin{array}{ll} \tilde{\mathbb{P}}(X_{i}(r_{i}^{k})=b_{i}^{k}\;|\;X_{i}(\tau_{k})=h,b_{i}^{k}\in ℰ) & =\underset{m\in {ℛ}_{b_{i}^{k}}}{\sum }{\tilde{\alpha }}_{mb_{i}^{k}}^{k_{r_{i}}}{\tilde{P}}_{hm}(\tau_{k},\tau_{k_{r_{i}}-1}) \end{array}$$

Substituting these results into the original expression we obtain:

(A13)
$$\begin{array}{ll} d_{gh,i}^{k} & =\frac{{\tilde{P}}_{a_{i}^{k}g}(l_{i}^{k},\tau_{k-1})\cdot {\tilde{\alpha }}_{gh}^{k}\cdot \sum_{m\in {ℛ}_{b_{i}^{k}}}{\tilde{\alpha }}_{mb_{i}^{k}}^{k_{r_{i}}}{\tilde{P}}_{hm}(\tau_{k},\tau_{k_{r_{i}}-1})}{\sum_{m\in {ℛ}_{b_{i}^{k}}}{\tilde{\alpha }}_{mb_{i}^{k}}^{k_{r_{i}}}{\tilde{P}}_{a_{i}^{k}m}(l_{i}^{k},\tau_{k_{r_{i}}-1})} \end{array}$$

## Appendix B Alternative Estimation Procedures

In this section, we discuss some alternative estimation procedures that can be used. The Nelson‐Aalen estimator can be derived by using three different extensions of the likelihood function (see Section IV.1.5 of [24]). It is also possible to consider different extensions of the likelihood in the multi‐state Markov setting, with the estimation procedure proposed in Section 2.3.1 following a “Poisson” approximation of the canonical multinomial extension described below.

### Canonical Multinomial Extension

The canonical extension of the univariate survival model for multi‐state Markov processes yields the following likelihood contribution per individual (See Section IV.4.1.5 of Andersen et al. [7]):

This has the interpretation of conditionally independent multinomially distributed numbers of jumps from each state g, such that $\left(dN_{gh,i}(t):h\neq g)∼\text{Mult}(Y_{g,i}(t),dA_{gh}(t)):h\neq g\right)$ given ${ℱ}_{t-,i}$. Note that for maximum likelihood estimation in the non‐parametric case, we cannot assume the cumulative intensities are absolutely continuous, and therefore it is unclear whether the intensities exist [24]. We therefore attempt to estimate the cumulative intensities directly, allowing the cumulative intensities to have both continuous and discrete components. Taking the logarithm we obtain:

$$\begin{array}{ll} \ell_{i}^{C} & =\int_{0}^{t_{if_{i}}^{*}}\underset{g\in ℋ}{\sum }\left\{\underset{h\neq g}{\sum }dN_{gh,i}(t)\log(Y_{g,i}(t)dA_{gh}(t))\right.\\ & \left.+\left(Y_{g,i}(t)-\underset{z\leftarrow g}{\sum }dN_{gz,i}(t)\right)\log\left(1-\underset{z\leftarrow g}{\sum }dA_{gz}(t)\right)\right\} \end{array}$$

Following the discussion in Section 2.3.1, we maximize over the class of cumulative intensity functions with jumps only on the observed event times, reducing the integrals to summations. Clearly, $dN_{gh}(t)=0$ whenever (g,h)∉𝒱, therefore we can replace $\sum_{g\in ℋ}\sum_{h\neq g}$ with $\sum_{(g,h)\in 𝒱}$:

$$\begin{array}{ll} \ell_{i}^{C} & =\overset{K}{\underset{k=1}{\sum }}\left\{\underset{(g,h)\in 𝒱}{\sum }d_{gh,i}^{k}\log(\alpha_{gh}^{k})\right.\\ & \left.+\underset{g\in ℋ}{\sum }\left(Y_{g,i}^{k}-\underset{z\leftarrow g}{\sum }d_{gz,i}^{k}\right)\log\left(1-\underset{z\leftarrow g}{\sum }\alpha_{gz}^{k}\right)\right\} \end{array}$$

For the E‐step (see Section 2.3.2), we calculate the expectation of the log‐likelihood given the observed information, transforming the $Y^{\prime }$s and $d^{\prime }$s into expected quantities (notationally introducing tildes above the letters, see Equation (10)). This yields the following for the expected value of the complete data log likelihood:

$$\begin{array}{ll} 𝔼[\ell^{C}\;|\;O,\tilde{\alpha }] & =\overset{K}{\underset{k=1}{\sum }}\left\{\underset{(g,h)\in 𝒱}{\sum }d_{gh}^{k}(\tilde{\alpha })\log(\alpha_{gh}^{k})\right.\\ & \left.+\underset{g\in ℋ}{\sum }\left(Y_{g}^{k}(\tilde{\alpha })-\underset{z\leftarrow g}{\sum }d_{gz}^{k}(\tilde{\alpha })\right)\log\left(1-\underset{z\leftarrow g}{\sum }\alpha_{gz}^{k}\right)\right\} \end{array}$$

For the M‐step, we take the derivative to obtain the score function:

$$\begin{array}{ll} \frac{d𝔼[\ell^{C}\;|\;O,\tilde{\alpha }]}{d\alpha_{gh}^{k}} & =\frac{d_{gh}^{k}(\tilde{\alpha })}{\alpha_{gh}^{k}}-\frac{Y_{g}^{k}(\tilde{\alpha })-\sum_{z\leftarrow g}d_{gz}^{k}(\tilde{\alpha })}{1-\sum_{z\leftarrow g}\alpha_{gz}^{k}} \end{array}$$

Equating to zero and factoring out $\alpha_{gh}^{k}$ we obtain:

(B1)
$$\begin{array}{ll} \alpha_{gh}^{k} & =\frac{d_{gh}^{k}(\tilde{\alpha })\left(1-\sum_{z\leftarrow g}\alpha_{gz}^{k}+\alpha_{gh}^{k}\right)}{Y_{g}^{k}(\tilde{\alpha })-\sum_{z\leftarrow g}d_{gz}^{k}(\tilde{\alpha })+d_{gh}^{k}(\tilde{\alpha })} \end{array}$$

Note that under the canonical multinomial extension the update rule for $\alpha_{gh}^{k}$ also depends on the values of jumps of the cumulative intensities for other transitions in the kth bin which have not been estimated yet, leading to a system of equations. For notational convenience, define:

$$\begin{array}{l} Q_{gh}^{k}=\frac{d_{gh}^{k}(\tilde{\alpha })}{Y_{g}^{k}(\tilde{\alpha })-\sum_{z\leftarrow g}d_{gz}^{k}(\tilde{\alpha })+d_{gh}^{k}(\tilde{\alpha })} \end{array}$$

Fix g∈ℋ and k∈{1,…,K} and consider the column vectors ${\vec{\alpha }}_{g}^{k}={\left[\alpha_{gh}^{k}\right]}_{\left\{h\leftarrow g\right\}}$ and ${\vec{Q}}_{g}^{k}={\left[Q_{gh}^{k}\right]}_{\left\{h\leftarrow g\right\}}$. Assume without loss of generality that the first and last state can be reached from state g in a single transition. Define:

$$\begin{array}{ll} {\vec{D}}_{g}^{k} & :=\text{diag}\left({\vec{Q}}_{g}^{k}\right)\\ & =\left(\begin{array}{ccc} \frac{d_{g1}^{k}(\tilde{\alpha })}{Y_{g}^{k}(\tilde{\alpha })-\sum_{z\leftarrow g}d_{gz}^{k}(\tilde{\alpha })+d_{g1}^{k}(\tilde{\alpha })} & \ldots & 0\\ \vdots & \ddots & \vdots \\ 0 & \ldots & \frac{d_{gH}^{k}(\tilde{\alpha })}{Y_{g}^{k}(\tilde{\alpha })-\sum_{z\leftarrow g}d_{gz}^{k}(\tilde{\alpha })+d_{gH}^{k}(\tilde{\alpha })} \end{array}\right) \end{array}$$

as well as the hollow matrix:

$$\begin{array}{ll} {\vec{M}}_{g} & :=\left(\begin{array}{cccc} 0 & 1 & \ldots & 1\\ 1 & 0 & \ldots & 1\\ \vdots & & \ddots & \vdots \\ 1 & 1 & \ldots & 0 \end{array}\right) \end{array}$$

with both matrices square with dimension equal to the number of state reachable from g: |{z←g}|. Then we can write the system of equations as follows:

$$\begin{array}{ll} {\vec{\alpha }}_{g}^{k} & ={\vec{D}}_{g}^{k}\left(\vec{1}-{\vec{M}}_{g}{\vec{\alpha }}_{g}^{k}\right) \end{array}$$

with $\vec{1}$ a column vector of ones. This equation can be solved by matrix inversion:

(B2)
$$\begin{array}{ll} {\vec{\alpha }}_{g}^{k} & ={\left(\vec{I}+{\vec{D}}_{g}^{k}{\vec{M}}_{g}\right)}^{-1}{\vec{D}}_{g}^{k}\vec{1} \end{array}$$

The matrix $\left(\vec{I}+{\vec{D}}_{g}^{k}{\vec{M}}_{g}\right)$ has entries 1 on the diagonal with the rest of the row filled with the same value:

$$\begin{array}{ll} \vec{I}+{\vec{D}}_{g}^{k}{\vec{M}}_{g} & =\\ & \;\left(\begin{array}{cccc} 1 & \frac{d_{g1}^{k}(\tilde{\alpha })}{Y_{g}^{k}(\tilde{\alpha })-\sum_{z\leftarrow g}d_{gz}^{k}(\tilde{\alpha })+d_{g1}^{k}(\tilde{\alpha })} & \cdots & \frac{d_{g1}^{k}(\tilde{\alpha })}{Y_{g}^{k}(\tilde{\alpha })-\sum_{z\leftarrow g}d_{gz}^{k}(\tilde{\alpha })+d_{g1}^{k}(\tilde{\alpha })}\\ \frac{d_{g2}^{k}(\tilde{\alpha })}{Y_{g}^{k}(\tilde{\alpha })-\sum_{z\leftarrow g}d_{gz}^{k}(\tilde{\alpha })+d_{g2}^{k}(\tilde{\alpha })} & 1 & \cdots & \frac{d_{g2}^{k}(\tilde{\alpha })}{Y_{g}^{k}(\tilde{\alpha })-\sum_{z\leftarrow g}d_{gz}^{k}(\tilde{\alpha })+d_{g2}^{k}(\tilde{\alpha })}\\ \vdots & \cdots & \ddots & \vdots \\ \frac{d_{gH}^{k}(\tilde{\alpha })}{Y_{g}^{k}(\tilde{\alpha })-\sum_{z\leftarrow g}d_{gz}^{k}(\tilde{\alpha })+d_{gH}^{k}(\tilde{\alpha })} & \frac{d_{gH}^{k}(\tilde{\alpha })}{Y_{g}^{k}(\tilde{\alpha })-\sum_{z\leftarrow g}d_{gz}^{k}(\tilde{\alpha })+d_{gH}^{k}(\tilde{\alpha })} & \cdots & 1 \end{array}\right) \end{array}$$

Note that the matrix $\left(I+{\vec{D}}_{g}^{k}{\vec{M}}_{g}\right)$ in general has full rank (and is therefore invertible). The matrix is clearly not invertible when $Y_{g}^{k}(\tilde{\alpha })=\sum_{s\leftarrow g}d_{gs}^{k}(\tilde{\alpha })$, as then all entries in the matrix will be equal to 1. This only happens when, according to the current estimates $\tilde{\alpha }$, there is no probability of staying in state g during $\left(\tau_{k}-,\tau_{k}\right]$.

### Multinoulli Extension

Another possible extension is given by Andersen and Ravn [7], yielding the following contribution to the likelihood per subject:

which has the interpretation of a multinomially distributed jump at each time point, such that $\left(dN_{gh,i}(t):(g,h)\in 𝒱\right)∼\text{Mult}(1,dA_{gh}(t):(g,h)\in 𝒱)$ given ${ℱ}_{t-,i}$. The multinomial distribution with a single draw is often referred to as a multinoulli distribution, therefore we will call this the “multinoulli” extension of the likelihood.

Taking very similar steps as in Appendix Section Canonical Multinomial Extension we are led to the following system of equations as maximizer of the expected complete‐data likelihood:

$$\begin{array}{ll} \alpha_{gh}^{k} & =\frac{d_{gh}^{k}(\tilde{\alpha })\left(1-\sum_{(r,s)\in 𝒱}\alpha_{rs}^{k}+\alpha_{gh}^{k}\right)}{n-\sum_{(r,s)\in 𝒱}d_{rs}^{k}(\tilde{\alpha })+d_{gh}^{k}(\tilde{\alpha })} \end{array}$$

Compared with Equation (B1), this expression has an n instead of $Y_{g}^{k}(\tilde{\alpha })$ and the summation is taken over all possible transitions instead of all possible transitions out of a single state g. Clearly, n is the expected number of subjects “at risk” of a transition at any time t, making the two approaches very similar. The multinomial approach considers each transition separately, whereas the multinoulli approach obtains estimates by considering all possible transitions together.

We can solve the resulting system of equations in a similar fashion. Define:

$$\begin{array}{l} Q_{gh}^{k\prime }=\frac{d_{gh}^{k}(\tilde{\alpha })}{n-\sum_{(r,s)\in 𝒱}d_{rs}^{k}(\tilde{\alpha })+d_{gh}^{k}(\tilde{\alpha })} \end{array}$$

Fix k∈{1,…,K} and consider the column vectors ${\vec{\alpha }}^{k}={\left[\alpha_{gh}^{k}\right]}_{\left\{(g,h)\in 𝒱\right\}}$ and ${\vec{Q}}^{k}={\left[Q_{gh}^{k^{\prime }}\right]}_{\left\{(g,h)\in 𝒱\right\}}$. Assume without loss of generality that the second state can be reached from the first and the second to last from the last state in a single transition. Define:

$$\begin{array}{ll} {\vec{D}}^{k} & :=\text{diag}\left({\vec{Q}}^{k}\right)\\ & =\left(\begin{array}{ccc} \frac{d_{12}^{k}(\tilde{\alpha })}{n-\sum_{(r,s)\in 𝒱}d_{rs}^{k}(\tilde{\alpha })+d_{12}^{k}(\tilde{\alpha })} & \ldots & 0\\ \vdots & \ddots & \vdots \\ 0 & \ldots & \frac{d_{H,H-1}^{k}(\tilde{\alpha })}{n-\sum_{(r,s)\in 𝒱}d_{rs}^{k}(\tilde{\alpha })+d_{H,H-1}^{k}(\tilde{\alpha })} \end{array}\right) \end{array}$$

as well as the hollow matrix:

$$\begin{array}{ll} \vec{M} & :=\left(\begin{array}{cccc} 0 & 1 & \ldots & 1\\ 1 & 0 & \ldots & 1\\ \vdots & & \ddots & \vdots \\ 1 & 1 & \ldots & 0 \end{array}\right) \end{array}$$

with both matrices square with dimension equal to the number of possible transitions in the model: |{(g,h)∈𝒱}|. Then we can write the system of equations as follows:

$$\begin{array}{ll} {\vec{\alpha }}^{k} & ={\vec{D}}^{k}\left(\vec{1}-\vec{M}{\vec{\alpha }}^{k}\right) \end{array}$$

with $\vec{1}$ a column vector of ones. This equation can be solved by matrix inversion:

(B3)
$$\begin{array}{ll} {\vec{\alpha }}^{k} & ={\left(\vec{I}+{\vec{D}}^{k}\vec{M}\right)}^{-1}{\vec{D}}^{k}\vec{1} \end{array}$$

The matrix $\left(\vec{I}+{\vec{D}}^{k}\vec{M}\right)$ has entries 1 on the diagonal with the rest of the row filled with the same value:

$$\begin{array}{ll} & \vec{I}+{\vec{D}}^{k}\vec{M}=\\ & \;\left(\begin{array}{cccc} 1 & \frac{d_{12}^{k}(\tilde{\alpha })}{n-\sum_{(r,s)\in 𝒱}d_{rs}^{k}(\tilde{\alpha })+d_{12}^{k}(\tilde{\alpha })} & \cdots & \frac{d_{12}^{k}(\tilde{\alpha })}{n-\sum_{(r,s)\in 𝒱}d_{rs}^{k}(\tilde{\alpha })+d_{12}^{k}(\tilde{\alpha })}\\ \frac{d_{13}^{k}(\tilde{\alpha })}{n-\sum_{(r,s)\in 𝒱}d_{rs}^{k}(\tilde{\alpha })+d_{13}^{k}(\tilde{\alpha })} & 1 & \cdots & \frac{d_{13}^{k}(\tilde{\alpha })}{n-\sum_{(r,s)\in 𝒱}d_{rs}^{k}(\tilde{\alpha })+d_{13}^{k}(\tilde{\alpha })}\\ \vdots & \cdots & \ddots & \vdots \\ \frac{d_{H,H-1}^{k}(\tilde{\alpha })}{n-\sum_{(r,s)\in 𝒱}d_{rs}^{k}(\tilde{\alpha })+d_{H,H-1}^{k}(\tilde{\alpha })} & \frac{d_{H,H-1}^{k}(\tilde{\alpha })}{n-\sum_{(r,s)\in 𝒱}d_{rs}^{k}(\tilde{\alpha })+d_{H,H-1}^{k}(\tilde{\alpha })} & \cdots & 1 \end{array}\right) \end{array}$$

Similarly, this matrix will be non‐invertible whenever $n=\sum_{(r,s)\in 𝒱}d_{rs}^{k}(\tilde{\alpha })$, and it therefore is impossible to stay in any of the states in the interval $\left(\tau_{k-1},\tau_{k}\right]$.
