# Supplementary material for: Nonparametric Estimation of Transition Intensities in Interval‐Censored Markov Multistate Models Without Loops
Source: Stat Med. 2025 Aug 15;44(18-19):e70225. doi: 10.1002/sim.70225 (PMC12355648; doi:10.1002/sim.70225)
Supplement: Supplementary file 1 — Data S1. Supporting Information [file SIM-44-0-s001.pdf]

Received: Added at production | Revised: Added at production | Accepted: Added at production

DOI: xxx/xxxx

SUPPLEMENTARY MATERIALS

Supplementary Materials to “Non-parametric estimation of transition intensities in interval censored Markov multi-state models without loops”

Daniel Gomon[1] | **Hein Putter**[1,2]

[1]Mathematical Institute, Leiden University, Leiden, the Netherlands

[2]Department of Biomedical Data Sciences, Leiden University Medical Centre, Leiden, the Netherlands

Correspondence
Daniel Gomon, Mathematical Institute, Leiden University, Leiden, the Netherlands
Email: d.gomon@math.leidenuniv.nl

Present address
Einsteinweg 55, 2333CC Leiden, the Netherlands

A | SIMULATION STUDY - FIGURES

This section presents the complete results concerning performance measures in the six considered scenarios.

 © Copyright Holder Name

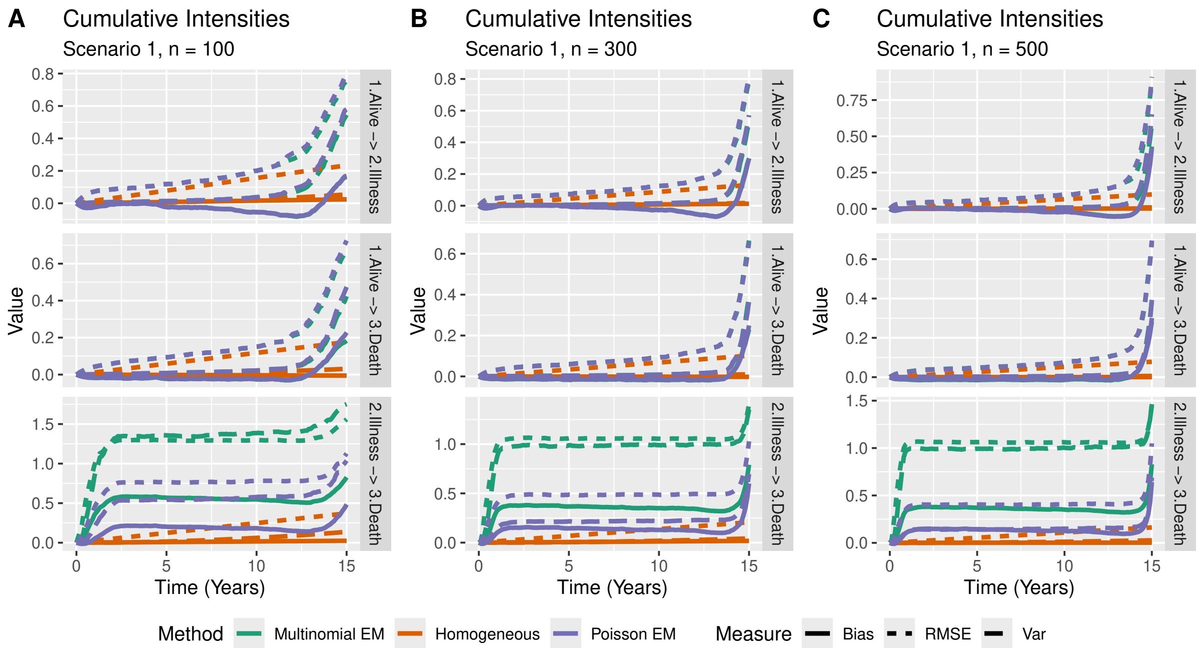

FIGURE 1 Bias, Variance and RMSE of cumulative intensities in scenario 1 for A) n = 100, B) n = 300, C) n = 500 samples in N = 1000 simulated data sets.

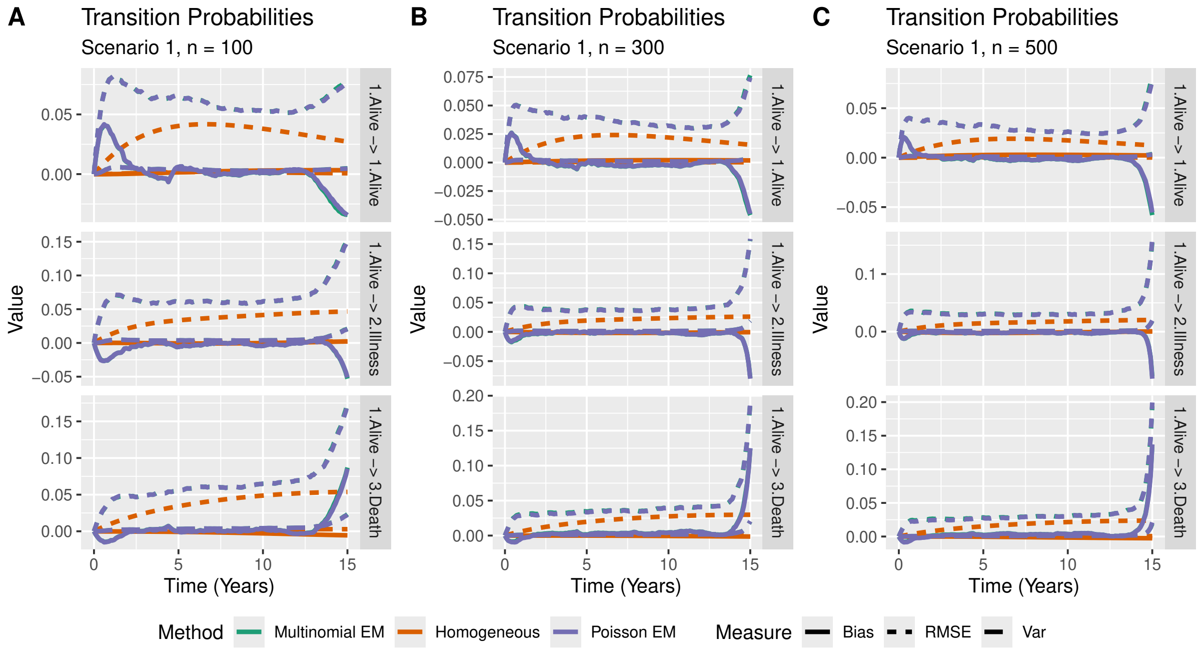

FIGURE 2 Bias, Variance and RMSE of transition probabilities in scenario 1 for A) n = 100, B) n = 300, C) n = 500 samples in N = 1000 simulated data sets.

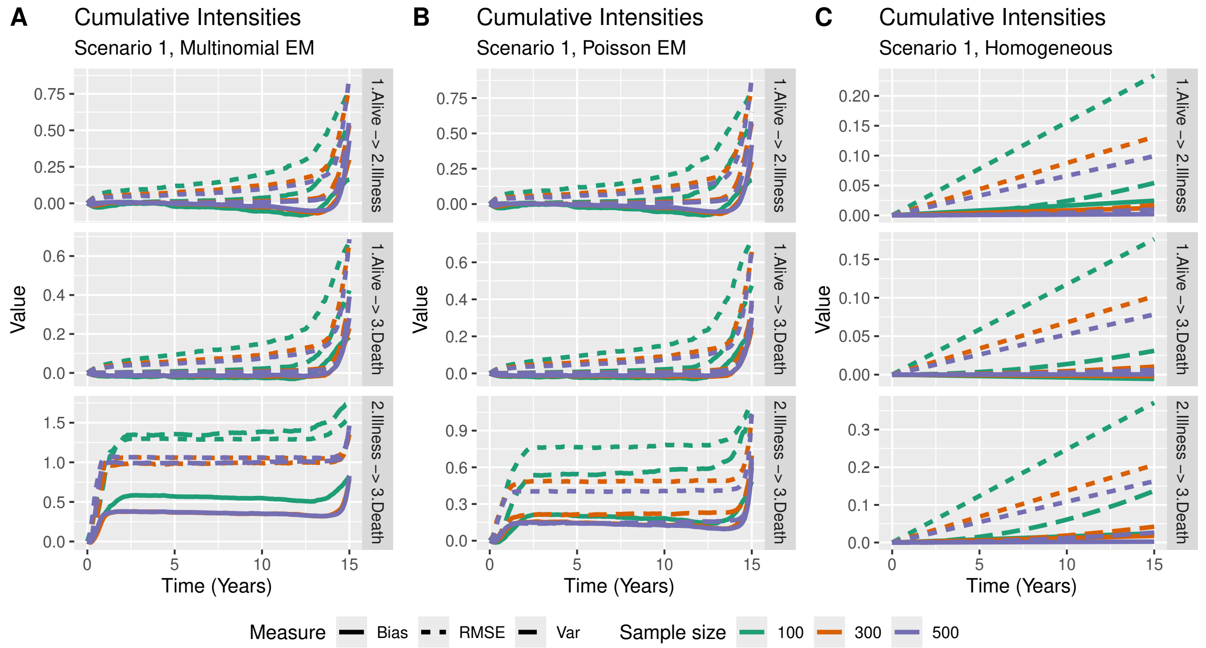

FIGURE 3 Bias, Variance and RMSE of cumulative intensities in scenario 1 for A) multinomial EM, B) Poisson EM, C) Time-homogeneous methods in $N = 1000$ simulated data sets.

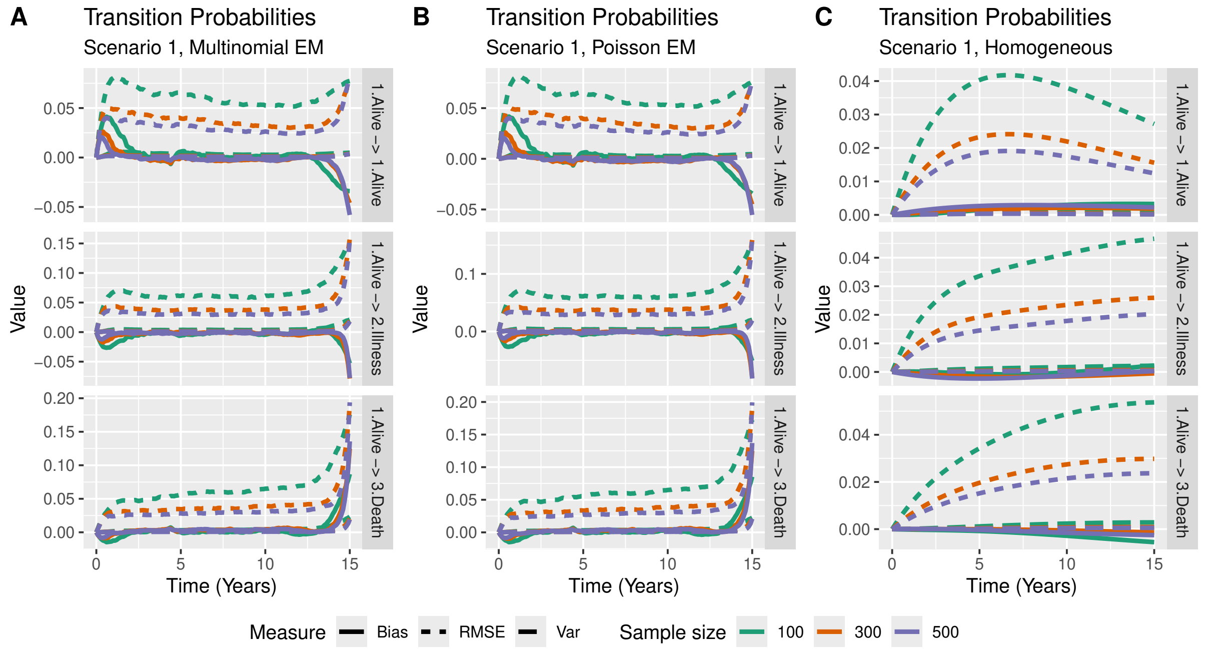

FIGURE 4 Bias, Variance and RMSE of transition probabilities in scenario 1 for A) multinomial EM, B) Poisson EM, C) Time-homogeneous methods in $N = 1000$ simulated data sets.

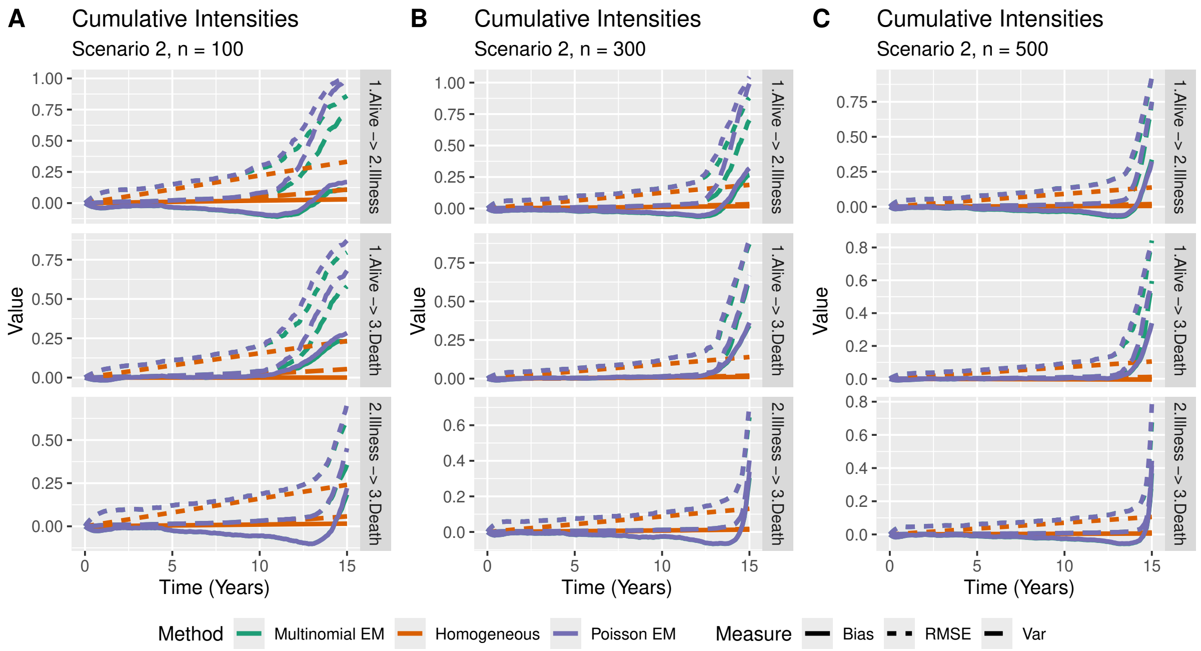

FIGURE 5 Bias, Variance and RMSE of cumulative intensities in scenario 2 for A) $n = 100$, B) $n = 300$, C) $n = 500$ samples in $N = 1000$ simulated data sets.

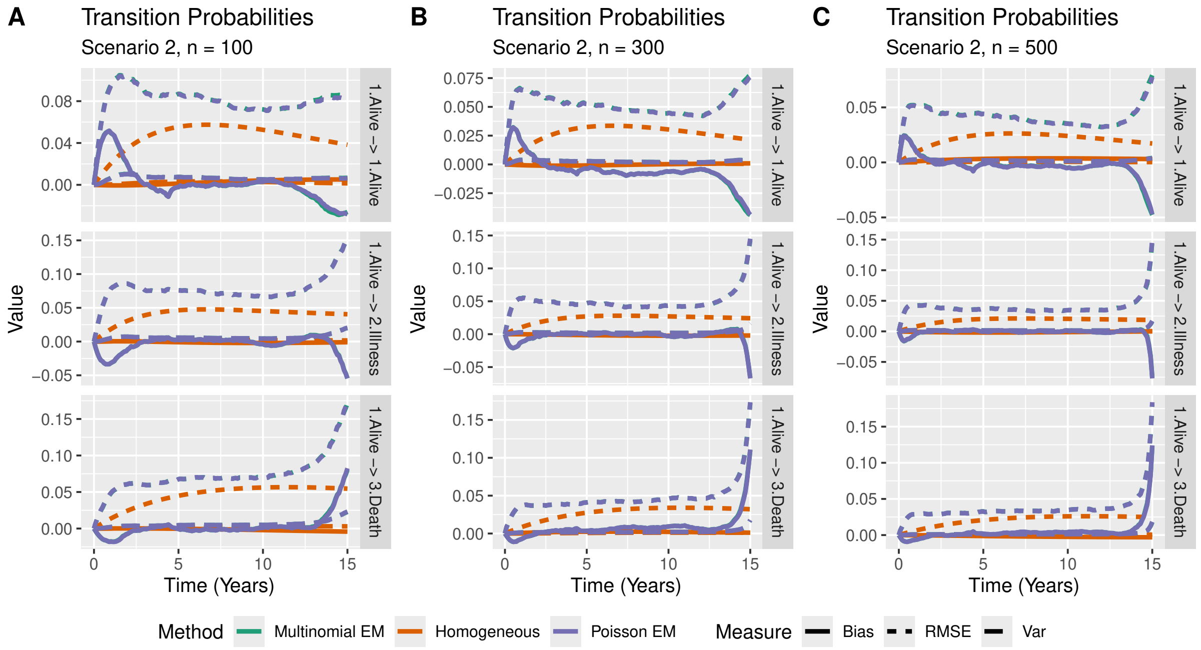

FIGURE 6 Bias, Variance and RMSE of transition probabilities in scenario 2 for A) $n = 100$, B) $n = 300$, C) $n = 500$ samples in $N = 1000$ simulated data sets.

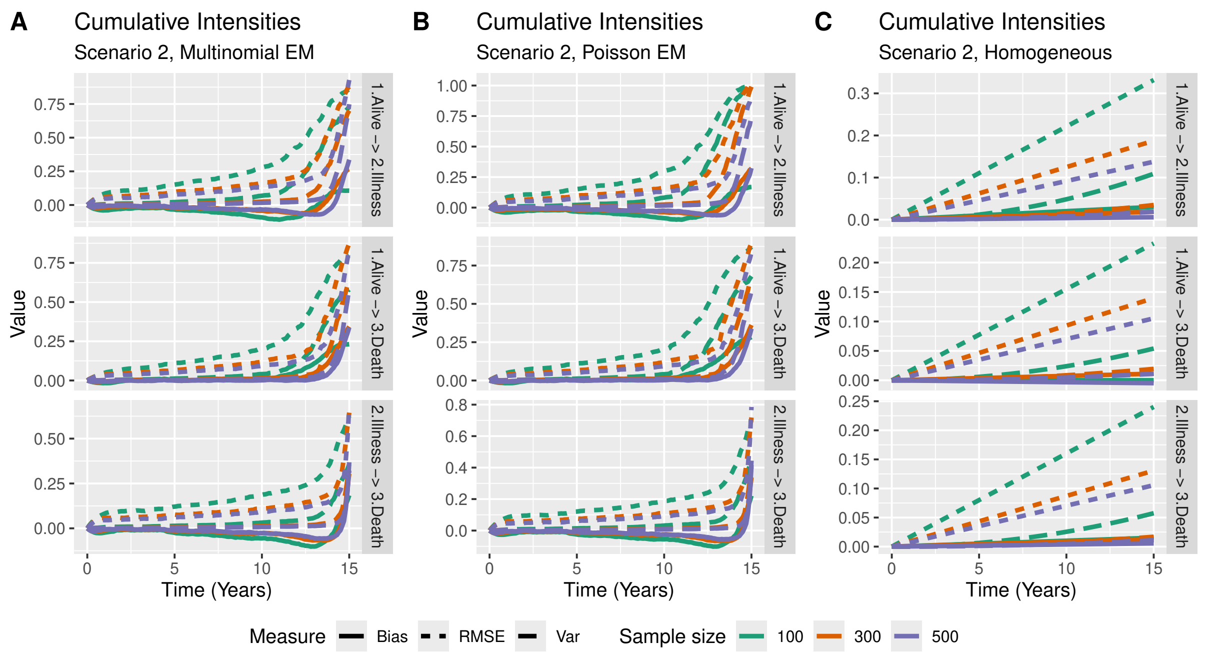

FIGURE 7 Bias, Variance and RMSE of cumulative intensities in scenario 2 for A) multinomial EM, B) Poisson EM, C) Time-homogeneous methods in $N = 1000$ simulated data sets.

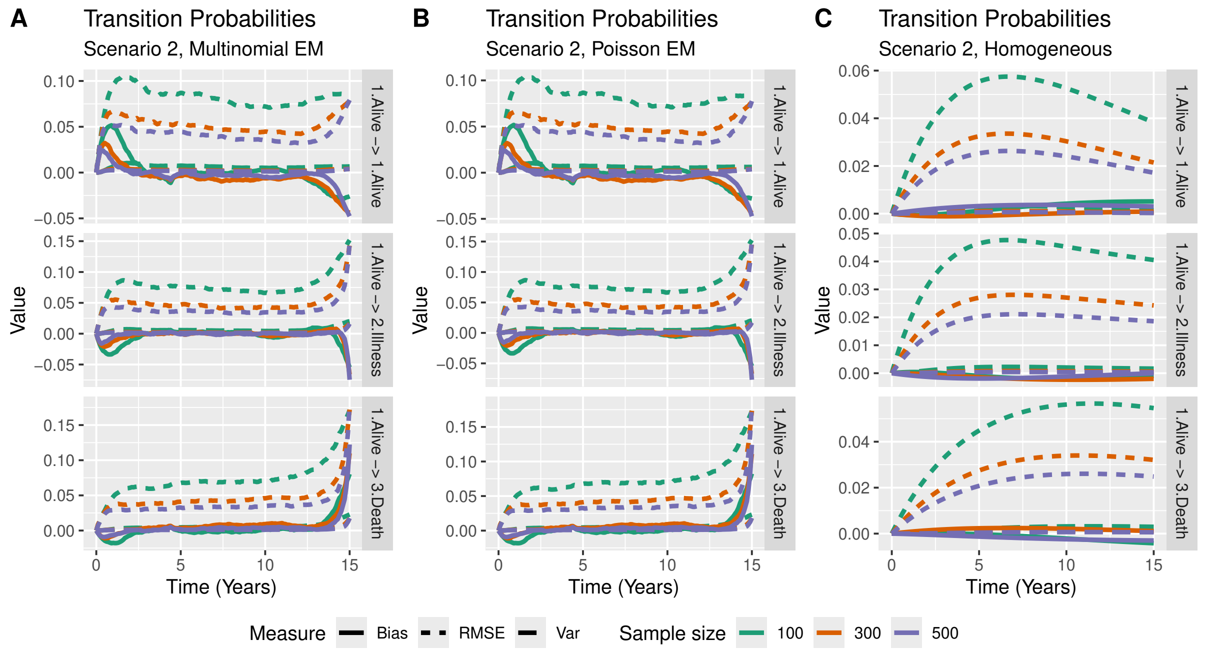

FIGURE 8 Bias, Variance and RMSE of transition probabilities in scenario 2 for A) multinomial EM, B) Poisson EM, C) Time-homogeneous methods in $N = 1000$ simulated data sets.

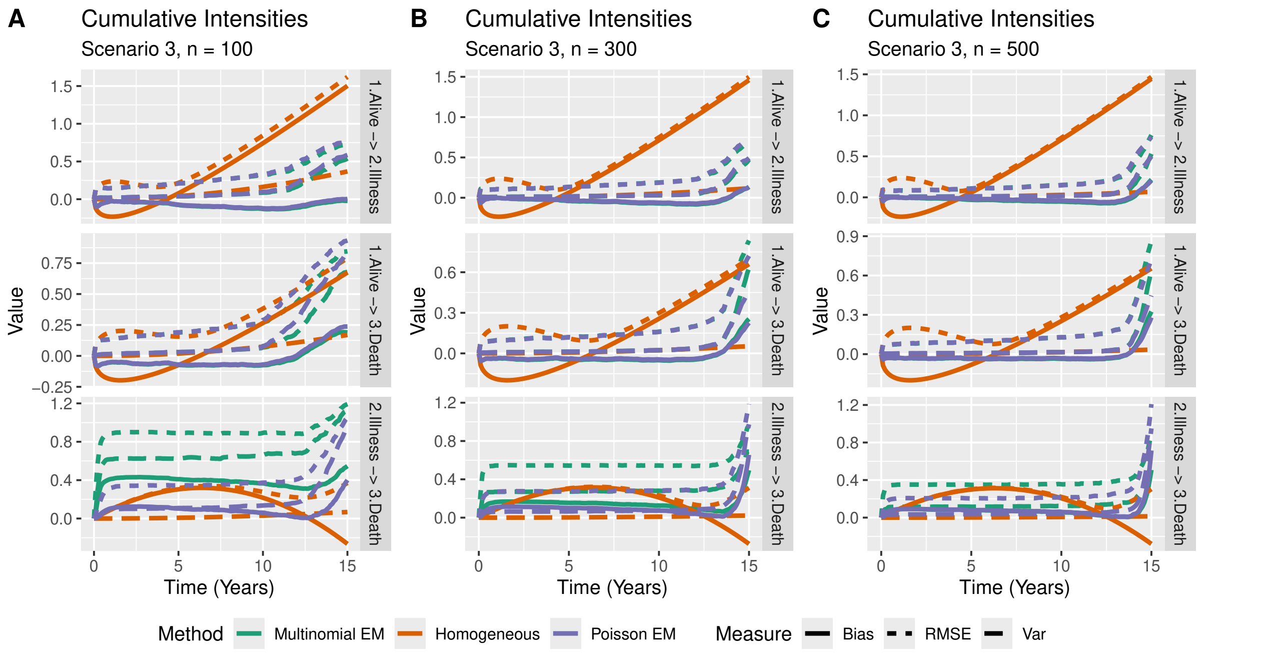

FIGURE 9 Bias, Variance and RMSE of cumulative intensities in scenario 3 for A) n = 100, B) n = 300, C) n = 500 samples in N = 1000 simulated data sets.

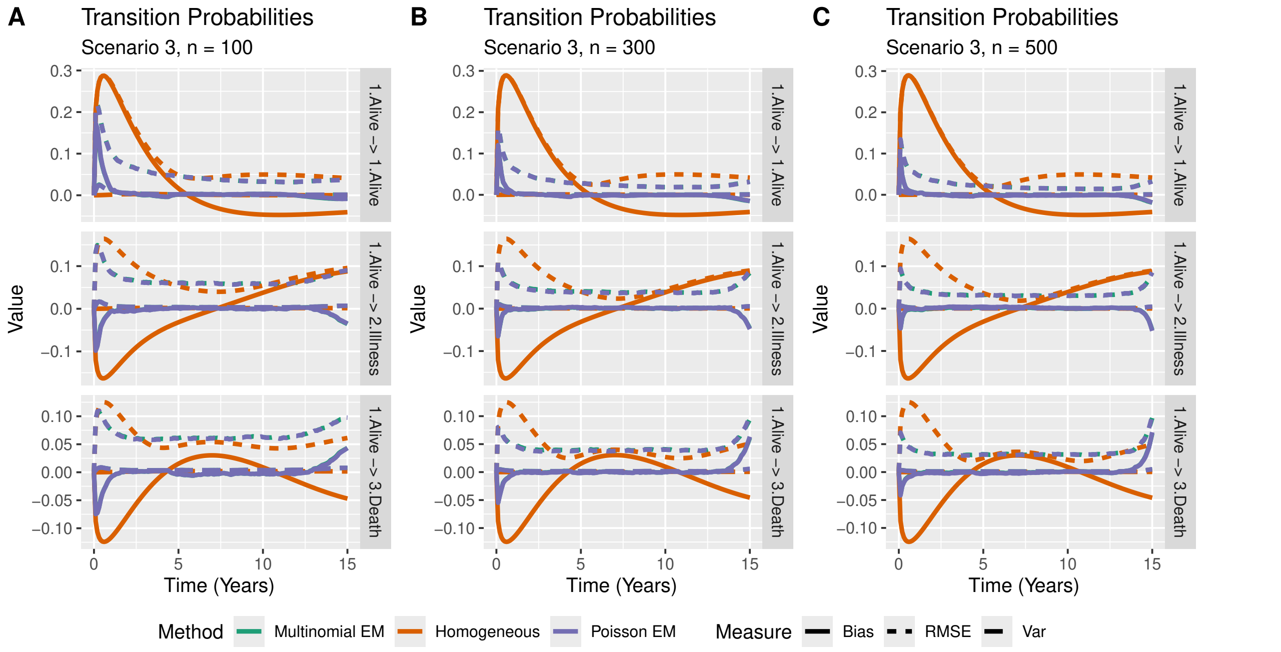

FIGURE 10 Bias, Variance and RMSE of transition probabilities in scenario 3 for A) n = 100, B) n = 300, C) n = 500 samples in N = 1000 simulated data sets.

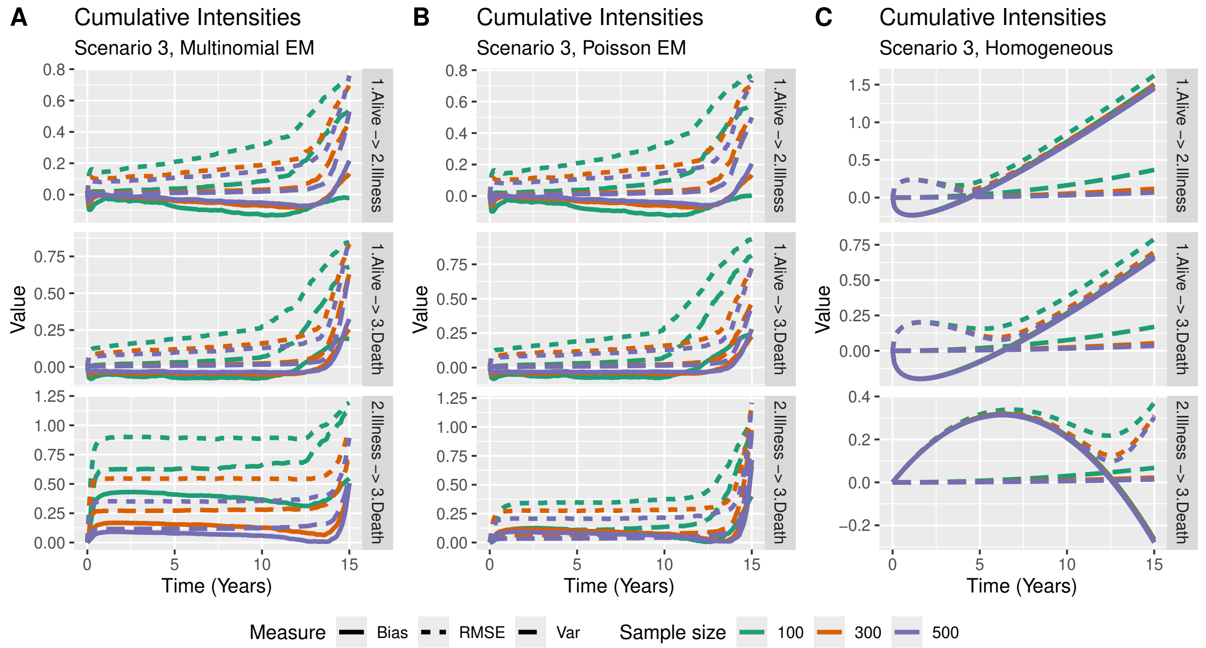

FIGURE 11 Bias, Variance and RMSE of cumulative intensities in scenario 3 for A) multinomial EM, B) Poisson EM, C) Time-homogeneous methods in $N = 1000$ simulated data sets.

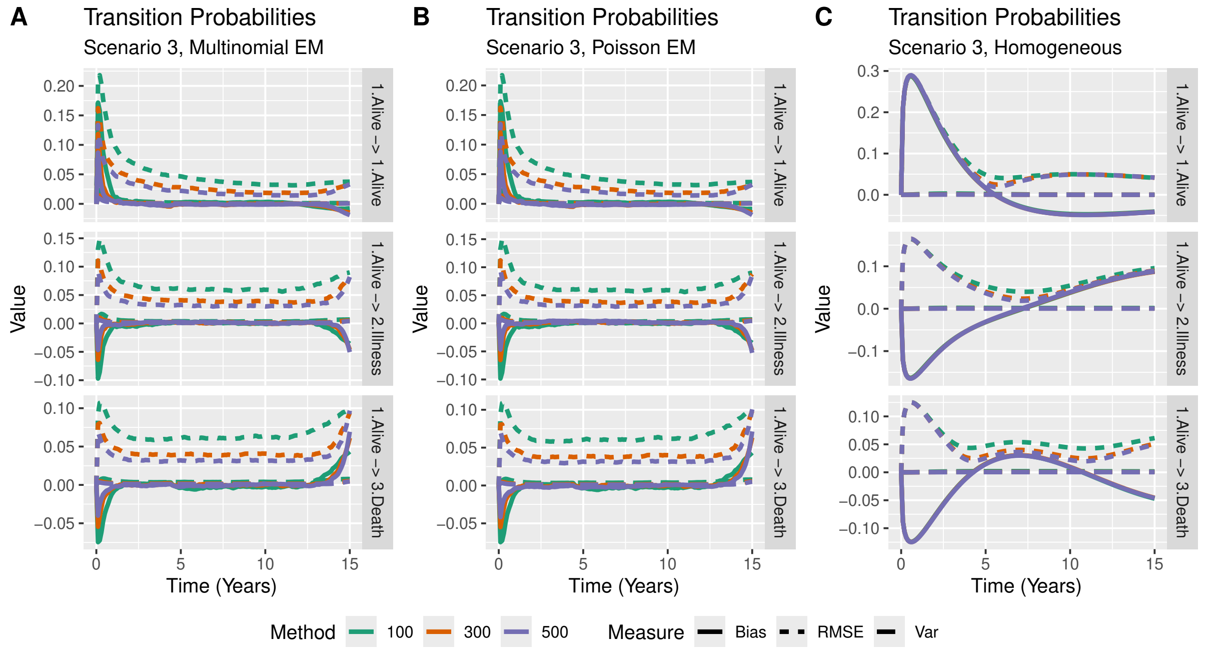

FIGURE 12 Bias, Variance and RMSE of transition probabilities in scenario 3 for A) multinomial EM, B) Poisson EM, C) Time-homogeneous methods in $N = 1000$ simulated data sets.

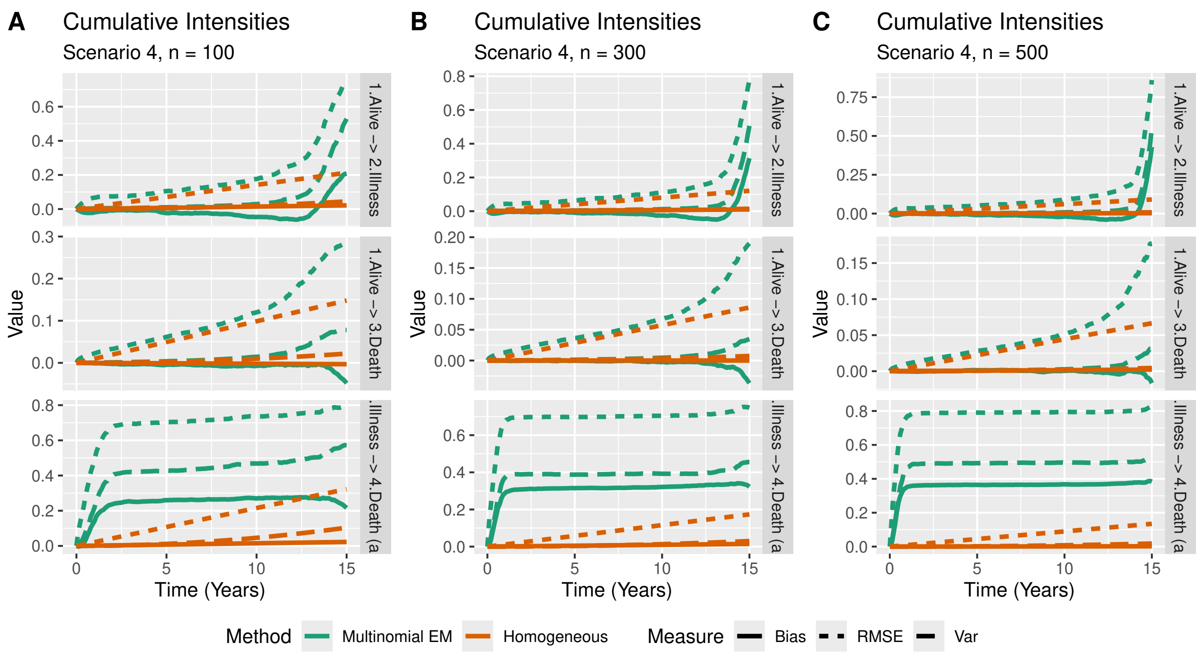

FIGURE 13 Bias, Variance and RMSE of cumulative intensities in scenario 4 for A) $n = 100$, B) $n = 300$, C) $n = 500$ samples in $N = 1000$ simulated data sets.

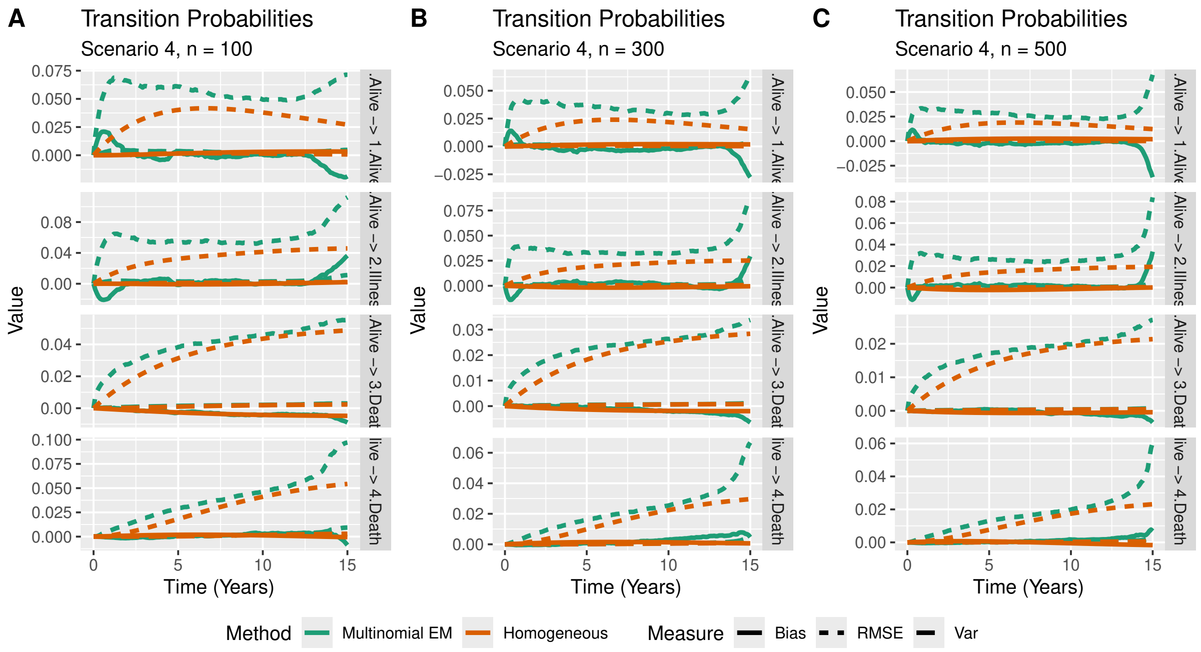

FIGURE 14 Bias, Variance and RMSE of transition probabilities in scenario 4 for A) $n = 100$, B) $n = 300$, C) $n = 500$ samples in $N = 1000$ simulated data sets.

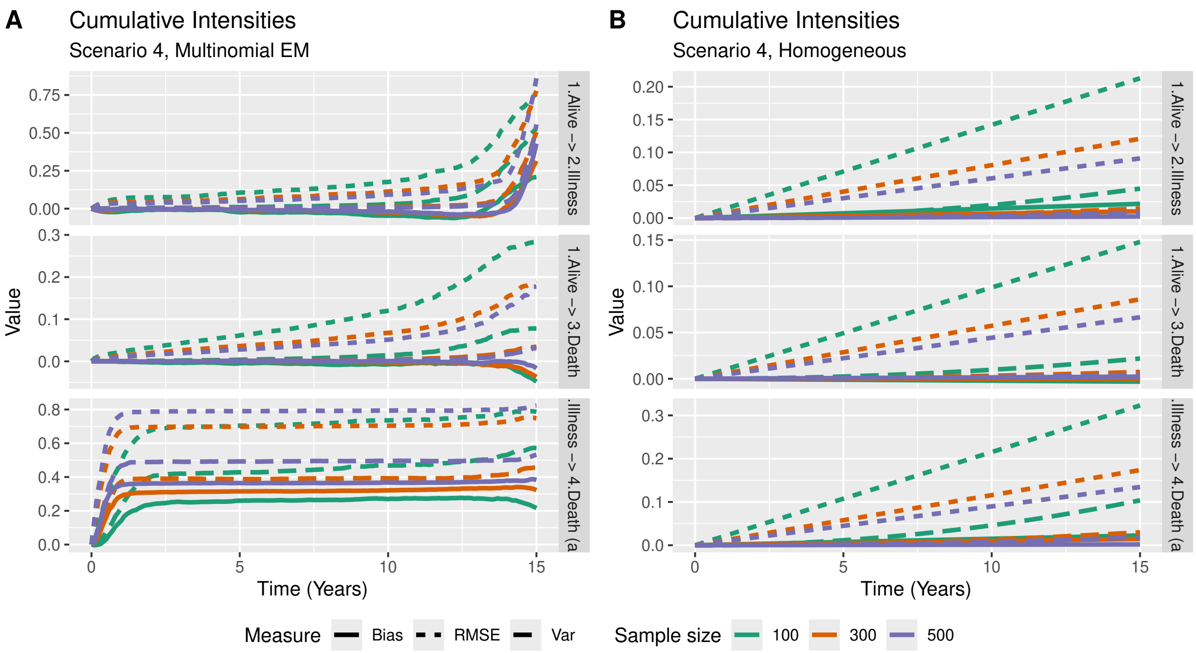

FIGURE 15 Bias, Variance and RMSE of cumulative intensities in scenario 4 for A) multinomial EM, B) Time-homogeneous methods in $N = 1000$ simulated data sets.

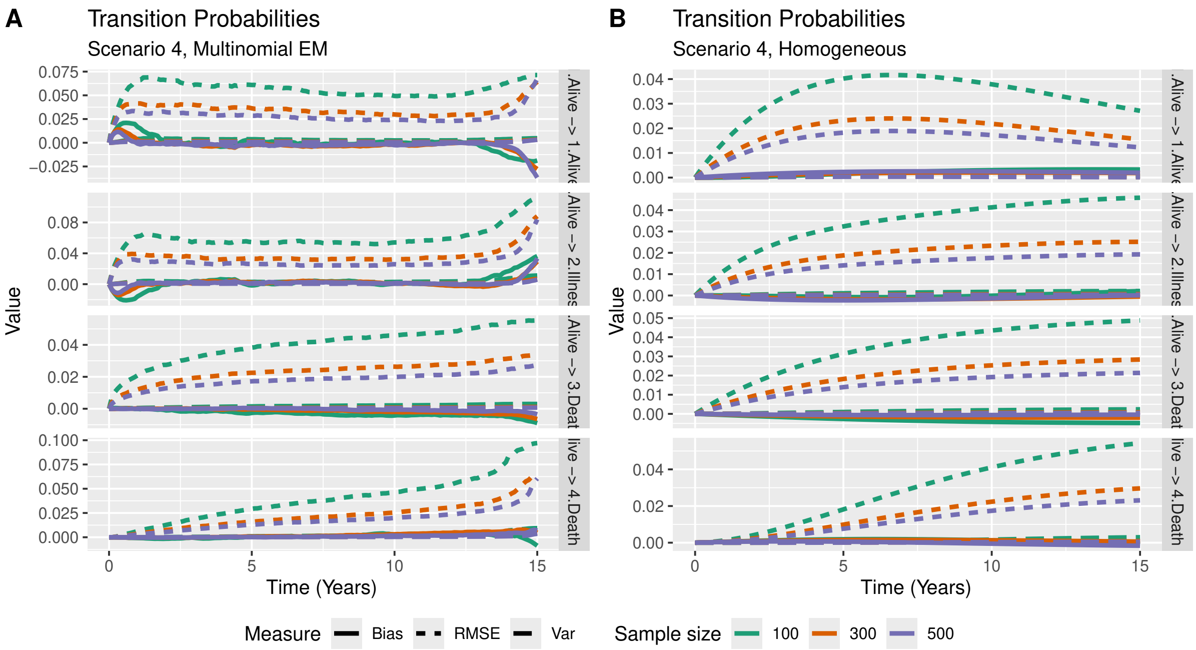

FIGURE 16 Bias, Variance and RMSE of transition probabilities in scenario 4 for A) multinomial EM, B) Poisson EM methods in $N = 1000$ simulated data sets.

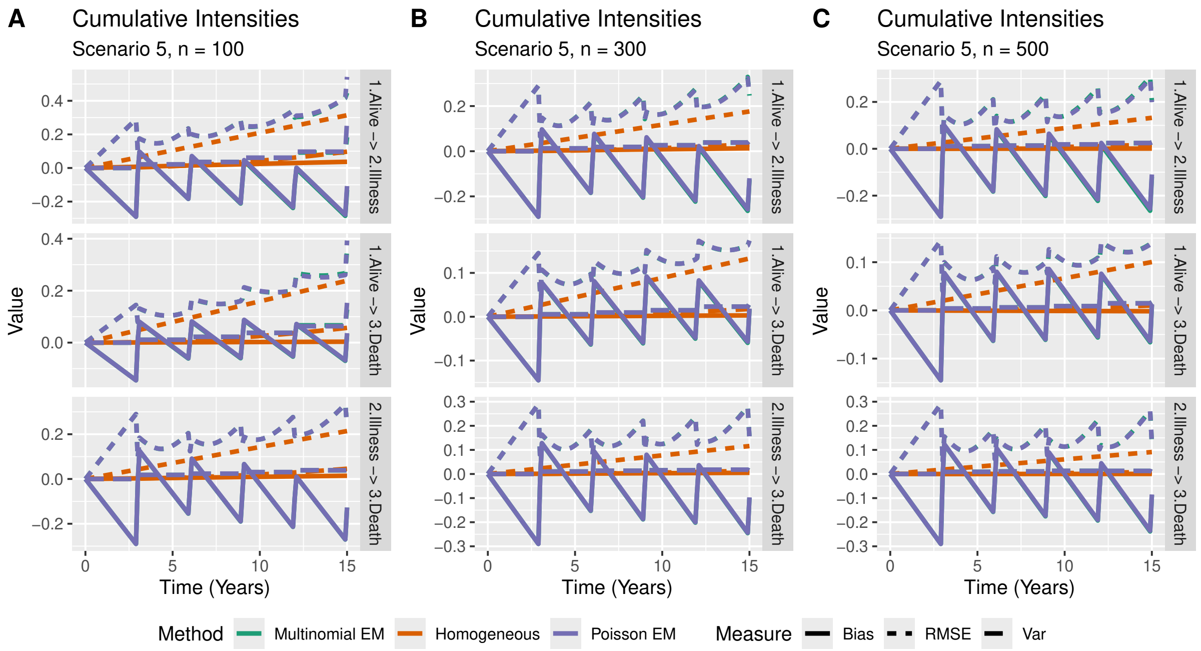

FIGURE 17 Bias, Variance and RMSE of cumulative intensities in scenario 5 for A) n = 100 B) n = 300 C) n = 500 samples in N = 1000 simulated data sets.

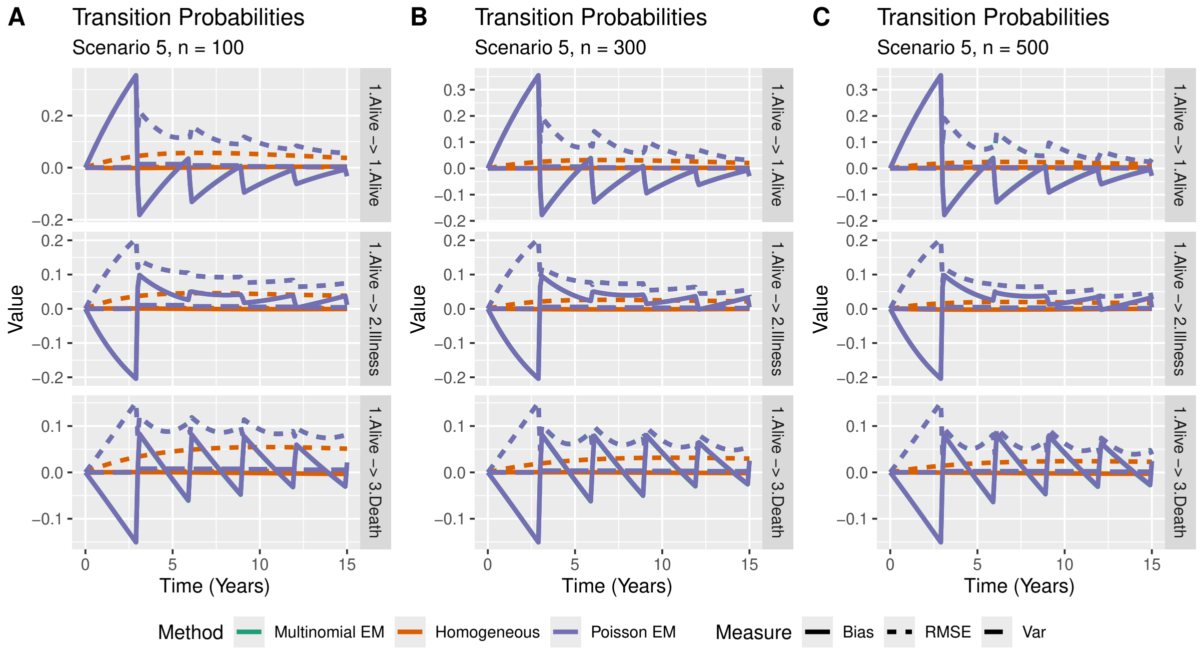

FIGURE 18 Bias, Variance and RMSE of transition probabilities in scenario 5 for A) n = 100 B) n = 300 C) n = 500 samples in N = 1000 simulated data sets.

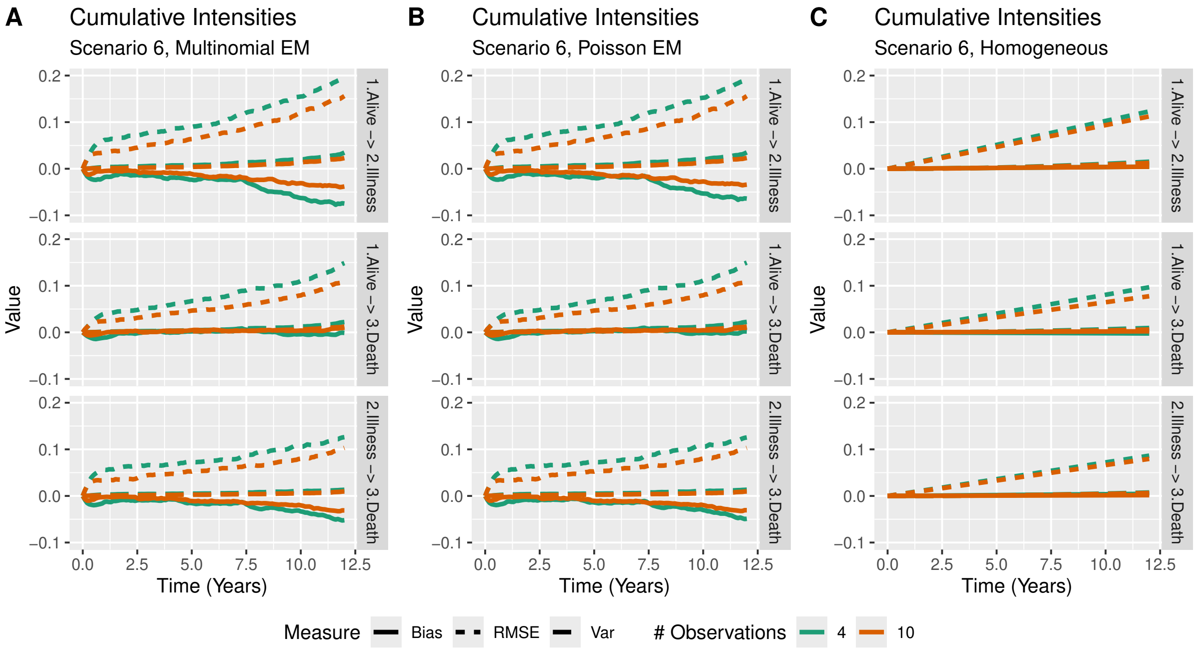

FIGURE 19 Bias, Variance and RMSE of cumulative intensities in scenario 6 for A) multinomial EM B) Poisson EM C) Time-homogeneous methods in $N = 1000$ simulated data sets for different numbers of observation times.

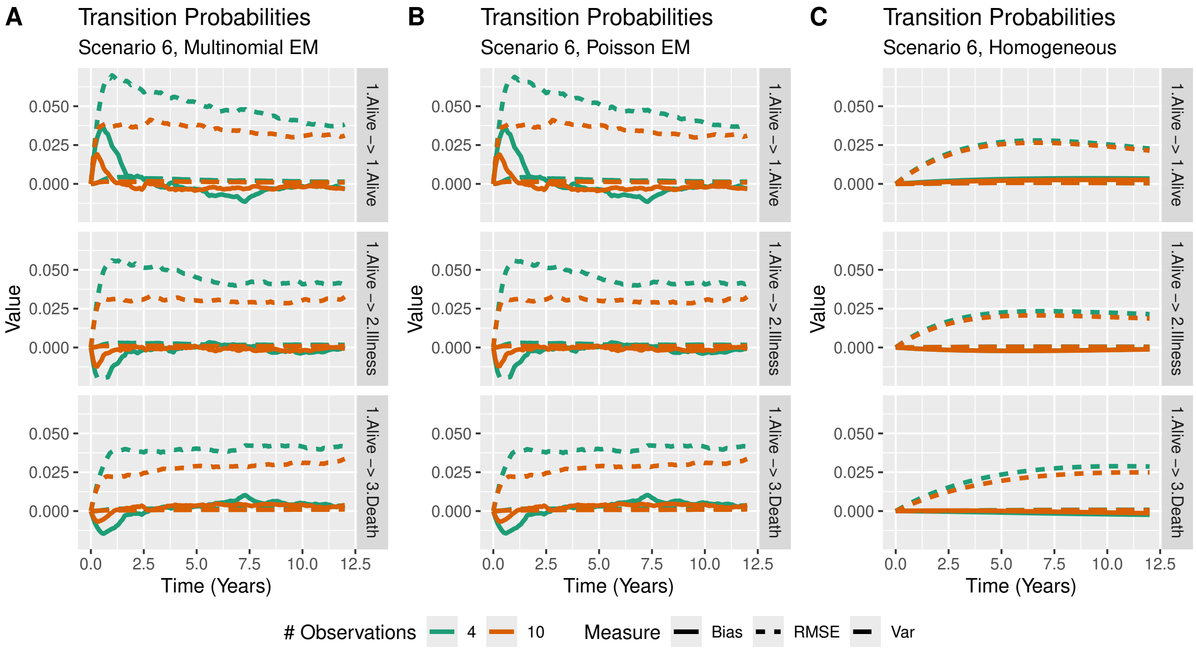

FIGURE 20 Bias, Variance and RMSE of transition probabilities in scenario 6 for A) multinomial EM B) Poisson EM C) Time-homogeneous methods in $N = 1000$ simulated data sets for different numbers of observation times.

B | LATENT POISSON EXPECTATION MAXIMISATION ALGORITHM

In this section, we loosely describe the EM algorithm for the NPMLE of interval-censored multi state data based on latent Poisson variables[1]. This Section therefore does not contain any original work.

Their EM algorithm is very similar to the multinomial EM algorithm, so most of the previous notation carries over. We do however need to introduce some new notation first, mainly for the latent Poisson variables. We consider the interval-censored multi state observed-data likelihood. Similar to above, the cumulative intensity function is assumed to be a right-continuous step function with jumps only at the unique observation times.

Fix i and j, then $[t_{i,j-1}, t_{ij}]$ is some observation interval with observed states $x_{i,j-1}$ and x_{ij}. Let w be the value such that $\tau_{w-1} = t_{i,j-1}$, and q the value such that $\tau_{w+q+1} = t_{ij}$. Then $t_{i,j-1} = \tau_{w-1} < \tau_w < \ldots < \tau_{w+q} < \tau_{w+q+1} = t_{ij}$ is a grid spanning the observation interval. A transition from $x_{i,j-1}$ to x_{ij} in the observation interval must have happened through one of the possible transition paths $(u_{w-1}, u_w, \ldots, u_{iw+q}, u_{w+q+1})$ with $u_w, \ldots, u_{w+q}$ the unobserved states at the corresponding times $\tau_w, \ldots, \tau_{w+q}$. For such a possible transition path, they define the event $V_i(u_w, \ldots, u_{w+q}, t_{i,j-1}, t_{ij}, x_{i,j-1}, x_{ij})$ through latent Poisson random variables $W^k_{gh,i}$ as follows. For $k = w, \ldots, w+q+1$, if $u_{k-1} \neq u_k$ then $W^k_{u_{k-1}u_k,i} > 0$ and $W^k_{u_{k-1}u',i} = 0$ for all $u' \neq u_{k-1}, u_k$. Else if $u_{k-1} = u_k$ we have that $W^k_{u_{k-1}u',i} = 0$ for all $u' \neq u_{k-1}$. They then define the event $Y_i(t_{i,j-1}, t_{ij}, x_{i,j-1}, x_{ij}) = \bigcup_{(u_w,\ldots,u_{w+q})\in\mathcal{A}_{w+q}} V_i(u_w, \ldots, u_{w+q}, t_{i,j-1}, t_{ij}, x_{i,j-1}, x_{ij})$ with $\mathcal{A}_{w+q}$ the set of possible transitions connecting $x_{i,j-1}$ and x_{ij}. They show that maximising the interval-censored multi state observed-data likelihood is the same as maximising the likelihood based on the observations $\mathcal{O}_i = \bigcap_{j=1}^{n_i} Y_i(t_{i,j-1}, t_{ij}, x_{i,j-1}, x_{ij})$.

They show that the complete-data log likelihood for these latent Poisson variables is given by:

$$\ell^{\mathrm{P}} = \sum_{i=1}^{n} \left(\sum_{k=1}^{K} \sum_{(g,h)\in\mathcal{V}} \mathbb{1}\{\tau_k \leq t_{in_i}\} \left[W^k_{gh,i} \log(\alpha^k_{gh}) - \alpha^k_{gh} - \log(W^k_{gh,i}!) \right] \right). \tag{1}$$

This likelihood is quite different compared to the complete-data log likelihood based on the multinomial distribution considered by us.

To determine an update rule for α^k_{gh}, they calculate the conditional expectation of the Poisson variables given the observed data and current estimates of the cumulative transition intensities:

$$\widetilde{\mathbb{E}}[W^k_{gh,i} \mid \mathrm{O}] = \mathbb{E}[W^k_{gh,i} \mid \mathrm{O}, \widetilde{\alpha}] = \frac{\sum_{g'\neq g} \widetilde{P}_{a^k_i g'}(l^k_i, \tau_k-) \widetilde{P}_{g' b^k_i}(\tau_k-, r^k_i)}{\widetilde{P}_{a^k_i b^k_i}(l^k_i, r^k_i)} \widetilde{\alpha}^k_{gh} \tag{2}$$

$$+ \frac{\widetilde{P}_{a^k_i g}(l^k_i, \tau_k-) \widetilde{\alpha}^k_{gh} \widetilde{P}_{hb^k_i}(\tau_k, r^k_i)}{\widetilde{P}_{a^k_i b^k_i}(l^k_i, r^k_i)} \exp\left(- \sum_{h' \leftarrow g, h' \neq h} \widetilde{\alpha}^k_{gh'} \right). \tag{3}$$

The update rule (M-step) in the EM algorithm is then given by:

$$\alpha^k_{gh} = \frac{\sum_{i=1}^{n} \mathbb{1}\{\tau_k \leq t_{i,n_i}\} \widetilde{\mathbb{E}}[W^k_{gh,i} \mid \mathrm{O}]}{\sum_{i=1}^{n} \mathbb{1}\{\tau_k \leq t_{i,n_i}\}}. \tag{4}$$

Contrary to our approach, they do not consider the KKT conditions to make sure that (sum of the) updated jumps in the intensities is bounded by zero and one in the M-step. In practice, this is unlikely to be an issue in non-parametric estimation, but might be problematic when covariates are included.

C | LATENT POISSON EM - INITIAL ESTIMATE DEPENDENCE

In this section, we show that the initial intensity estimates for transitions out of non-absorbing states that are not covered by the initial state cannot be changed by the latent Poisson approach.

Consider $g, h \in \mathcal{H}$ such that the transition $g \to h$ is possible. Assume that no subject starts at time 0 in state g. Consider the first bin $[0, \tau_1]$ and a single subject $i \in \{1, \ldots, n\}$. The contribution of this subject to the value of α^1_{gh} is given by Equation (2):

$$\begin{aligned}\widetilde{E}[W^1_{gh,i} \mid \mathrm{O}] = &\frac{\sum_{g' \neq g} \widetilde{P}_{a^1_i g'}(l^1_i, \tau_1-)\widetilde{P}_{g' b^1_i}(\tau_1-, r^1_i)}{\widetilde{P}_{a^1_i b^1_i}(l^1_i, r^1_i)} \widetilde{\alpha}^1_{gh} \\ &+ \frac{\widetilde{P}_{a^1_i g}(l^1_i, \tau_1-)\widetilde{\alpha}^1_{gh}\widetilde{P}_{hb^1_i}(\tau_1, r^1_i)}{\widetilde{P}_{a^1_i b^1_i}(l^1_i, r^1_i)} \exp\left(-\sum_{h' \leftarrow g, h' \neq h} \widetilde{\alpha}^1_{gh'}\right).\end{aligned}$$

Clearly $l^1_i = \tau_1 - = 0$, so we obtain:

$$\begin{aligned}\widetilde{E}[W^1_{gh,i} \mid \mathrm{O}] = &\frac{\sum_{g' \neq g} \widetilde{P}_{a^1_i g'}(0,0)\widetilde{P}_{g' b^1_i}(0, r^1_i)}{\widetilde{P}_{a^1_i b^1_i}(0, r^1_i)} \widetilde{\alpha}^1_{gh} \\ &+ \frac{\widetilde{P}_{a^1_i g}(0,0)\widetilde{\alpha}^1_{gh}\widetilde{P}_{hb^1_i}(\tau_1, r^1_i)}{\widetilde{P}_{a^1_i b^1_i}(0, r^1_i)} \exp\left(-\sum_{h' \leftarrow g, h' \neq h} \widetilde{\alpha}^1_{gh'}\right).\end{aligned}$$

Note that $\widetilde{P}_{a^1_i g'}(0,0)$ can only be non-zero when $g' = a^1_i$. The summation in the numerator of the first term therefore only yields a positive contribution when $g' = a^1_i$. As we assumed that no subjects start in state g, we must have that $g \neq a^1_i$ and therefore the second term is 0. We obtain:

$$\widetilde{E}[W^1_{gh,i} \mid \mathrm{O}] = \frac{\sum_{g' \neq g} \widetilde{P}_{a^1_i g'}(0,0)\widetilde{P}_{g' b^1_i}(0, r^1_i)}{\widetilde{P}_{a^1_i b^1_i}(0, r^1_i)} \widetilde{\alpha}^1_{gh} = \frac{\widetilde{P}_{a^1_i a^1_i}(0,0)\widetilde{P}_{a^1_i b^1_i}(0, r^1_i)}{\widetilde{P}_{a^1_i b^1_i}(0, r^1_i)} \widetilde{\alpha}^1_{gh} = \widetilde{\alpha}^1_{gh}.$$

Therefore the contribution of all subjects is the same at each iteration. From Equation (4) we then find that the estimate of $\widetilde{\alpha}^1_{gh}$ does not change over any iteration and the final estimate will simply be the initial estimate. The result shown here can also be extended to any $W^k_{gh,i}$ where no subject can be in state g before time τ_k. The summation in the numerator of the first term of Equation (2) represents the probability of reaching b^k_i through any path that does not go through state g at time τ_k-. The denominator represents the probability of reaching b^k_i through any path. As we assume that no subject can be in state g at time τ_k-, the numerator and denominator are equal and therefore the first term simply becomes $\widetilde{\alpha}^k_{gh}$. The second term represents the probability of making the $g \to h$ transition in the bin $(\tau_k-, \tau_k]$, but as we assumed no subject can be in state g at time τ_k- this probability clearly is zero. This means that the estimate for the jumps in the transition intensities cannot be changed for an interval where there is zero probability to be in state g at the start of that interval.

REFERENCES

1. Gu Y, Zeng D, Heiss G, Lin DY. Maximum likelihood estimation for semiparametric regression models with interval-censored multistate data. *Biometrika.* 2024;111(3):971–988.
